# New‐Era Polymer Thermoelectrics: Material Innovations, Doping Frontiers, Decoupling Strategies, and Unconventional Applications

**DOI:** 10.1002/adma.202522912

**Published:** 2026-04-09

**Authors:** Suhao Wang

**Affiliations:** ^1^ Unité de Dynamique et Structure des Matériaux Moléculaires Université du Littoral Côte d'Opale Dunkerque France

**Keywords:** chemical doping, ion‐exchange doping, polaron, polymer thermoelectrics, Seebeck coefficient, thermoelectric materials

## Abstract

The field of polymer thermoelectrics has undergone transformative development in recent years, marked not only by addressing the conventional performance disparity between p‐type and n‐type polymers, but also by innovations in doping methodologies, new strategies for suppressing dopant‐induced disorder, approaches for overcoming the inherent thermoelectric trade‐off, and advancements in achieving better ambient and thermal stability, as well as their variety of new applications. Collectively, these pivotal advances have brought the field into a new era. This review focuses on the recent development of high‐performance polymers that bridge the long‐standing performance gap, introduces doping frontiers for more highly optimized thermoelectric properties, and discusses sophisticated strategies for decoupling electronic and thermal transport. Moreover, this work unlocks the emerging understanding of the degradation mechanisms of doped polymers that enables the design of materials with superior ambient and thermal robustness, and showcases the burgeoning applications of thermoelectric polymers in unconventional domains.

## Graphical abstract

The field of polymer thermoelectrics is entering a new era, featuring breakthroughs in addressing the conventional performance disparity between p‐type and n‐type polymers, pioneering doping frontiers, and sophisticated decoupling strategies. This review explores innovations in molecular design and superior stabilities, bridging the gap from fundamental mechanisms to unconventional applications.

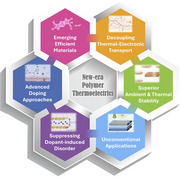

## 1 Introduction

**FIGURE 1:**
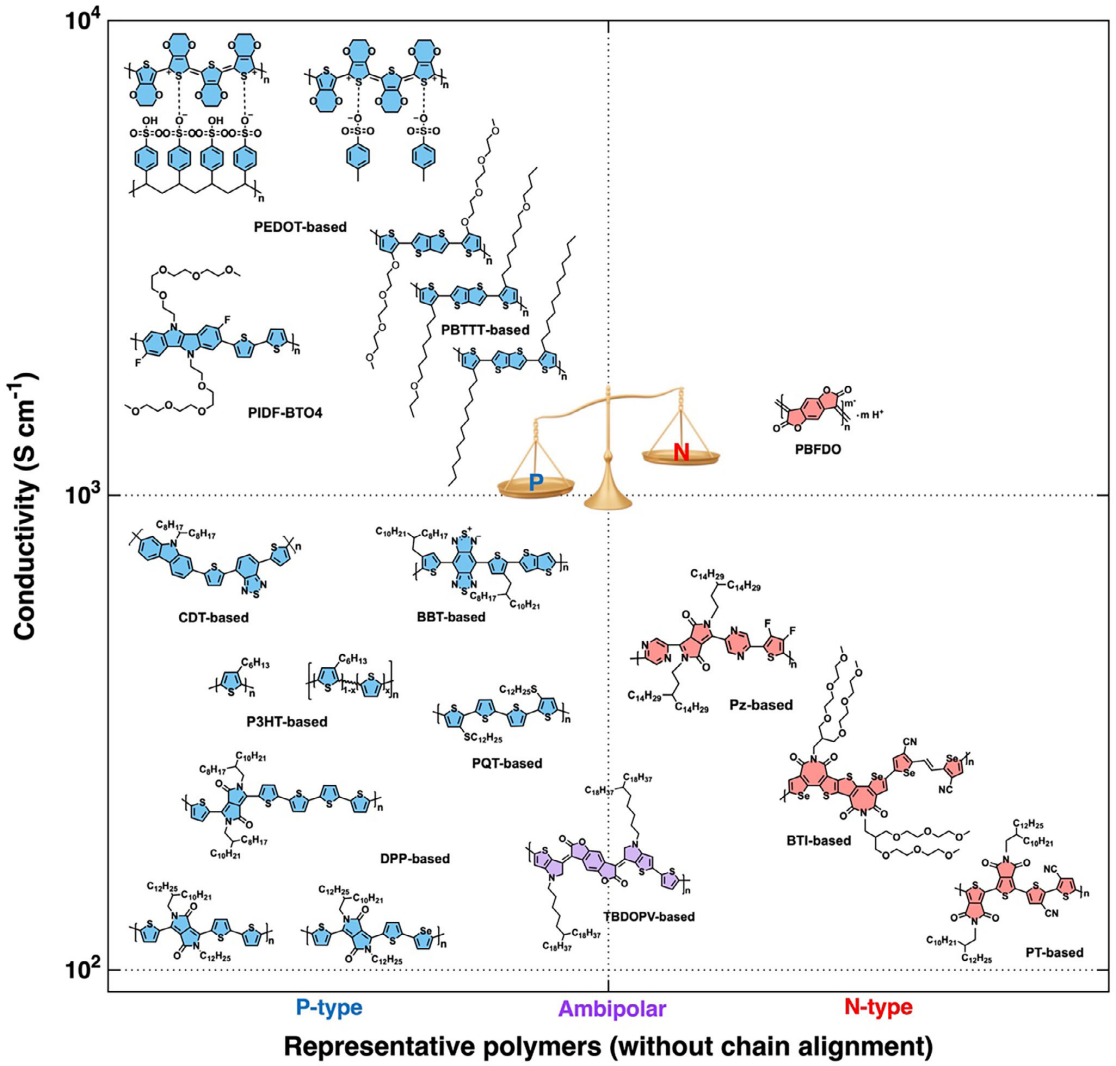
Representative highly conductive p‐type and n‐type thermoelectric polymers with electrical conductivities exceeding 100 S cm^−1^.

Thermoelectric (TE) materials, which allow for the quiet conversion of waste heat into valuable electricity without any moving components, have garnered exceptional attention over the past half‐century [1, 2, 3]. Instead of being used for substantial energy generation [4], thermoelectric generators (TEGs) play an essential role in capturing various forms of waste heat and enabling a variety of emerging applications [5, 6]. For several decades, inorganic materials, owing to their notable TE performance, have been the focus of scientific research and commercialization [7, 8]. Since the 2010s, conjugated polymers have surfaced as a promising category of TE materials [9, 10], thanks to their various advantages over their inorganic counterparts [11], including inherently low thermal conductivity [12], solution processability [13], solution‐state aggregation‐mediated assembly [14], mechanical flexibility [15, 16, 17, 18], printability [19, 20], the abundance of constituent elements, as well as diverse synthesis methodologies [21, 22, 23]. During the past fifteen years, considerable progress has been made in the development of polymer thermoelectrics, including significant performance enhancements [24, 25], and the advancement of various unconventional applications in sensing [26, 27], the Internet of Things (IoT) [28, 29], and flexible and wearable devices [30, 31]. Despite these advances, there have been several historical hurdles in the field retarding the development and commercial implementation of polymer thermoelectrics. i): Building complementary circuits necessitates both p‐type (hole‐transporting) [32] and n‐type (electron‐transporting) [33, 34, 35, 36] materials; however, the advancement of n‐type materials lags behind their p‐type counterparts. A variety of p‐type polymers exhibit electrical conductivities over 1000 S cm^−1^, such as PEDOT‐based polymers [37, 38, 39], PBTTT [40, 41, 42], and other p‐type polymers [43] (Figure 1). In contrast, the progress of n‐type polymers has lagged considerably [34], with the majority exhibiting electrical conductivities below 100 S cm^−1^, and in most cases below 10 S cm^−1^ [9]. ii) The doping efficiency of organic semiconducting materials is often limited by the formation of charge transfer complexes [44], and regionally controlling the doping process has been a challenge [45]. iii) Dopant‐induced disorder severely disrupts the microstructure of the doped films [46, 47], often resulting in deteriorated charge transport properties and lower thermoelectric performance [48]. iv) The trade‐off between the Seebeck coefficient and electrical conductivity remains a significant obstacle, limiting the maximum achievable TE performance for over a decade [44, 45, 49]. v) Poor ambient stability and low thermal stability of organic‐doped films significantly impede the commercialization of polymer thermoelectrics [50, 51].

Encouragingly, recent efforts in the field of polymer thermoelectrics, particularly over the preceding half‐decade, have resulted in substantial advances in addressing the aforementioned challenges. i) The synthesis of unconventional materials has resulted in a significant enhancement in the performance of n‐type polymers, with the extraordinarily conductive poly(benzodifurandione) (PBFDO) as the prime example, showing electrical conductivities exceeding 1000 S cm^−1^ [52, 53, 54]; several other systems, including pyrazine (Pz) based polymers [55, 56], polythiophene (PT) based polymers [57, 58], and bithiophene imide (BTI) based and analogous polymers [59, 60], exhibit electrical conductivities exceeding 100 S cm^−1^, (Figure 1) Furthermore, efficient ambipolar polymers have been synthesized and characterized, allowing for simultaneous p‐doping and n‐doping within a single material, leading to electrical conductivities exceeding 100 S cm^−1^ for both carrier types [61]. ii) Doping efficiency has been enhanced by developing novel doping methodologies, such as hybrid doping [62], solvent combination doping [43], as well as innovative doping strategies, such as ion‐exchange doping [41, 63], and catalytic‐based doping [64, 65]. Moreover, a new doping and fabrication strategy was recently developed to achieve regionally controlled doping with remarkable spatial resolution [66]. iii) Dopant‐induced disorder can be largely suppressed by several strategies, including designing disorder‐tolerant polymers [55], side‐chain cleavage of polymers [67, 68, 69], rational engineering of polymer‐dopant interaction [56, 70], and modulation doping [42]. iv) Novel strategies, including polymeric multi‐heterojunction (PMHJ) [71] and molecular‐force‐driven anisotropy (MFDA) [72], among other strategies, have been developed to address the trade‐off between electrical conductivity, the Seebeck coefficient, and the thermal conductivity, resulting in significantly enhanced thermoelectric properties of conjugated polymers, giving rise to a high p‐type dimensionless figure of merit (ZT) exceeding 1.0 [71, 73], and an n‐type ZT approaching 0.5 [74, 75]. v) Several effective strategies have been designed to enhance the ambient stability and thermal stability of polymer thermoelectrics, including the development of new doping methods (e.g., photocatalytic doping) [65], and the advancement of stable dopants (e.g., Lewis‐paired dopant complexes) [76]. In addition, significant progress has been made in the use of thermoelectric polymers for wearable fibers and elastomers [77, 78]. Especially noteworthy are the latest developments in n‐type thermoelectric elastomers [74, 79]. All the aforementioned advancements have brought the field of polymer thermoelectrics to a new era.

While several seminal review articles have established the fundamentals and the early development of organic thermoelectrics, the field has recently reached a critical inflection point. There is an urgent need for a conceptual framework that captures this rapid progress and the corresponding paradigm shifts. To provide a clear path forward, this review is structured specifically to address the aforementioned five historic hurdles limiting the potential of polymer thermoelectrics. Consequently, rather than presenting a simple chronological survey or covering the literature in its entirety, the following sections synthesize the recent breakthroughs that are systematically overcoming these obstacles.

Section 2 focuses on the first hurdle by highlighting emerging state‐of‐the‐art polymers, specifically the breakthrough in n‐type materials and novel ambipolar thermoelectric polymers, along with their design principles. Subsequently, Section 3 addresses the low doping efficiency (the second hurdle) by introducing an advanced doping toolkit and unconventional next‐generation doping approaches and physics, including ion‐exchange, proton‐coupled electron transfer, and photocatalytic doping, among others. Section 4 focuses on overcoming the third hurdle via a systematic analysis of unconventional strategies for suppressing dopant‐induced disorder. To address the trade‐off described in hurdle 4, Section 5 discusses morphology and orientation control of thermoelectric polymers for decoupling the thermal and electronic transport. The critical challenge of long‐term robustness, the fifth hurdle, is detailed in Section 6, which offers emerging mechanistic understanding of degradation and design rules for ambient‐ and thermal‐stable doping. Examples of unconventional applications for thermoelectric polymers are presented in Section 7. Subsequently, the translation from these breakthroughs in laboratory‐based thin films to robust, commercialized devices is still facing a series of bottlenecks, which are discussed in Section 8. Finally, the review concludes with insights into the future outlook and remaining challenges.

## 2 The Development of Thermoelectric Polymers

### 2.1 Recent Progress in p‐Type Thermoelectric Polymers

**FIGURE 2:**
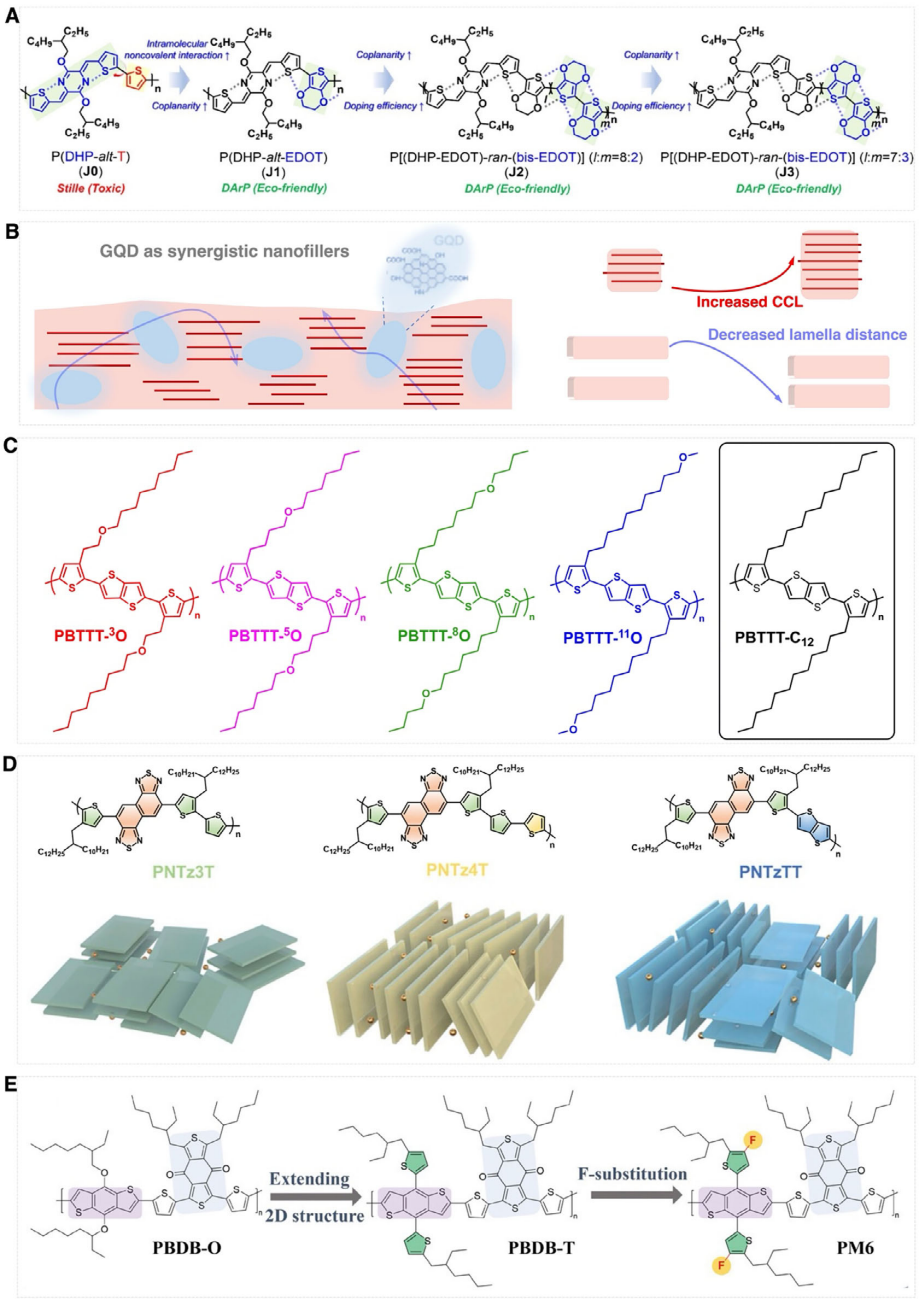
(A) Molecular design strategies of DHP‐based alternating (alt) and random (ran) copolymers synthesized via Stille or DArP. Reproduced under the terms of the CC‐BY Creative Commons Attribution 4.0 International license (https://creativecommons.org/licenses/by/4.0) [80]. Copyright 2025, The Authors, published by Wiley‐VCH. (B) Schematic illustration of GQD as synergetic nanofillers for PEDOT‐PSS. Concept based on ref [83]. (C) Chemical structures of the ether‐based PBTTT‐^x^O and PBTTT‐C12. Reproduced under the terms of the CC‐BY Creative Commons Attribution 4.0 International license (https://creativecommons.org/licenses/by/4.0) [86]. Copyright 2024, Royal Society of Chemistry. (D) Chemical structures of NTz‐based D‐A conjugated polymers and illustration of their corresponding orientation in doped films. Adapted under the terms of the CC‐BY Creative Commons Attribution 4.0 International license (https://creativecommons.org/licenses/by/4.0) [94]. Copyright 2024, The Authors, published by Wiley‐VCH. (E) Illustration of the evolution of chemical structures via a two‐step design rule for PBDB‐O, PBDB‐T, and PM6. Adapted under the terms of the CC‐BY Creative Commons Attribution 4.0 International license (https://creativecommons.org/licenses/by/4.0) [95]. Copyright 2024, The Authors, published by Wiley‐VCH.

P‐type conjugated polymers have been well developed, demonstrating remarkable thermoelectric performance. The most recent development is mainly based on the various molecular designing strategies for the enhancement of conventional high‐performance thermoelectric polymers, such as with ethylenedioxythiophene (EDOT)‐based polymers, PBTTT, and their derivatives. For instance, quinoidal dihydropyrazine (DHP) has been used as a promising building block to copolymerize with EDOT, allowing for the synthesis of a series of p‐type polymers with open‐shell electronic configurations [80] (Figure 2A). These random copolymers can be processed in eco‐friendly solvents, and the resulting films possess extended conjugation, facilitating effective intramolecular charge transport. On the other hand, the compact 𝜋–𝜋 stacking distance of 3.38 Å promotes effective intermolecular charge transport. Moreover, the use of spaced‐out alkyl pendant groups on DHP enhances the diffusion of the dopants, resulting in efficient doping. As a result, the polymers exhibit exceptional electrical conductivities of up to 1680 ±120 S cm^−1^ [80]. Recently, several works have been focusing on enhancing the thermoelectric properties of PEDOT‐PSS. One of the strategies is based on a sequential post‐treatment protocol, which involves H_2_SO_4_, NaOH, dimethyl sulfoxide (DMSO), and finally the electron donor 4‐(1,3‐dimethyl‐2,3‐dihydro‐1H‐benzoimidazol‐2‐yl)phenyl)dimethylamine (N‐DMBI) [81]. Hereby, the treatment with N‐DMBI is essential as it not only induces a partial dedoping of the PEDOT‐PSS, but also forms 𝜋–𝜋 interactions with the conjugated PEDOT backbone, which splits off the lower polaron level and thus pushes the Fermi level up, resulting in a significant boost in Seebeck coefficient up to 64.1 µV K^−1^ and a high power factor of 765.1 µW m^−1^ K^−2^. In a similar fashion, treating PEDOT‐PSS films with a tetrathiafulvalene (TTF) solution can elevate the Fermi level of PEDOT, thus markedly boosting the Seebeck coefficient without sacrificing the electrical conductivity, resulting in electrical conductivities up to 2254 S cm^−1^ and large Seebeck coefficient of 71 µV K^−1^, and thus a high power factor of 1285 µW m^−1^ K^−2^ and a high ZT value of 0.8 [82]. Very recently, a novel three‐step method was employed to treat PEDOT‐PSS films, with each step playing a distinct role but with all steps working in concert [83] (Figure 2B). Specifically, methanol enhances the film's molecular packing, and formic acid removes the insulating PSS for higher electrical conductivity, whereas graphene quantum dots act as nanofillers that bridge neighbouring PEDOT domains and simultaneously are responsible for interfacial energy filtering for a higher Seebeck coefficient. Consequently, the as‐treated films exhibit a high‐power factor of 382.6 µW m^−1^ K^−2^. In particular, the readers are referred to two comprehensive reviews on the recent development in optimizing the PEDOT‐based thermoelectrics [39, 84]. Leclerc et al. replaced the linear alkyl n‐C_12_ on PBTTT with a chain of the same length that includes an ether n‐C_7_OC_4_, and a polar derivative polymer is synthesized, PBTTT‐^8^O [85]. This side‐chain substitution maintains the synthetic ease and ambient stability of the alkylated reference polymer, but strengthens the structural order of the polymer backbones. Consequently, when solution‐doped with F_6_TCNNQ, the PBTTT‐^8^O exhibits a high electrical conductivity of 1100 ± 200 S cm^−1^, significantly higher than that of PBTTT‐C_12_ (130 ± 50 S cm^−1^). When aligned via a rubbing technique, both polymers exhibited an increase in conductivity of over 10‐fold [85]. Recently, the same team synthesized a series of PBTTT derivatives by adjusting the position of the single oxygen atom along the ether function, allowing for a systematic investigation of the impact of side chains on the crystallinity and thermoelectric performance [86] (Figure 2C). Owing to the hyperconjugation effect, the crystallinity increases as the oxygen atom is moved further from the PBTTT backbone, and finally, the PBTTT‐^8^O exhibits the highest thermoelectric performance. Generally, side‐chain engineering is a powerful tool for tuning the solid‐state microstructure, molecular packing, doping behavior and the thermoelectric performance of conjugated polymers [87, 88]. More details can be found in a recent review article [89]. Furthermore, by dissolving tris(pentafluorophenyl)borane (BCF) in the non‐polar aliphatic solvent hexane, efficient diffusion of BCF into PBTTT films can be achieved, resulting in a high doping efficiency and remarkable thermoelectric performance (230 S cm^−1^ and 140 µW m^−1^ K^−2^, respectively) [90]. Moreover, when BCF was used to dope the PBTTT‐derived polymer, poly(2‐(4,4′‐bis(2‐methoxyethoxy)‐5′‐methyl‐[2,2′‐bithiophen]‐5‐yl)‐5‐methylthieno‐[3,2‐b]thiophene (pgBTTT), a high doping level was achieved, resulting in a high electrical conductivity of 2180 S cm^−1^ and a maximum power factor of 223 µWm^−1^ K^2^ [91]. Notably, adding a trivial amount of nucleating agent N,N′‐(1,4‐phenyl)diisonicotinamide (PDA) into PBTTT resulted in significantly enhanced degree of crystallization, and the PDA‐treated PBTTT can be doped to a high electrical conductivity of 1894 S cm^−1^ and maximum power factor of 176 µWm^−1^ K^2^ [92]. Remarkably, by blending high‐ and low‐molecular‐weight chains of PBTTT‐C_12_, tie‐chain incorporation has been well controlled, and its essential role has been examined utilizing a variety of spectroscopic and microstructural approaches. Interestingly, although no significant changes were detected in the morphology or structural order of these crystalline domains, a significant improvement in thermoelectric performance (4180 S cm^−1^ and 173 µW m^−1^ K^−2^) was achieved, as a result of the tie chains aiding charge transport across grain boundaries [93].

Donor engineering in donor‐acceptor polymers can have a significant effect on the thermoelectric performance of donor–acceptor (D–A) polymers. Recently, the Naphthobisthiadiazole (NTz) acceptor was copolymerized with three different donors, dialkylated terthiophene (3T), dialkylated quaterthiophene (4T), and dialkylated bisthienyl thienothiophene (2T‐TT), yielding the corresponding copolymers, PNTz3T, PNTz4T, and PNTzTT [94] (Figure 2D). Compared to the other two polymers, PNTz4T allows for an edge‐on packing, facilitating in‐plane charge transport and efficient dopant intercalation, which ensures a higher doping level. Consequently, PNTz4T exhibits the highest thermoelectric performance among the series [94]. Enhancing backbone planarity can help extend 2D structures for better electrical performance, whereas fluorination of the backbone can disrupt the homogeneous electrostatic potential across the polymer backbone, leading to controlled induction of energetic disorder and, thus, an enhanced Seebeck coefficient.(Figure 2E) Remarkably, the combination of extended 2D structures and fluorination of the polymer backbones results in an enhancement of power factor as much as 32 times [95]. Furthermore, Kim et al. synthesized a new p‐type conjugated polymer poly(2‐([2,2'‐bi‐thiophen]‐5‐yl)‐3,8‐difluoro‐5,10‐bis(5‐octylpen‐tadecyl)‐5,10‐dihydroindolo [3,2‐b] indole) (PIDF‐BTO4), exhibiting an excellent electrical conductivity over 2000 S cm^−1^ after solution doping [43]. Since this work involves the development of novel solution‐doping approaches, more details will be discussed in Section 3. Generally speaking, a comprehensive review previously summarized various p‐type conjugated polymers [9]. Herein, the readers are referred to a recent review that systematically summarizes the molecular engineering for p‐type TE polymers [32]. In Section 2.2, an emphasis will be placed on the recent development of the most impressive n‐type TE materials.

### 2.2 Emerging Efficient n‐type Thermoelectric Polymers

#### 2.2.1 The State‐of‐the‐art Highly Conductive n‐Type PBFDO

**FIGURE 3:**
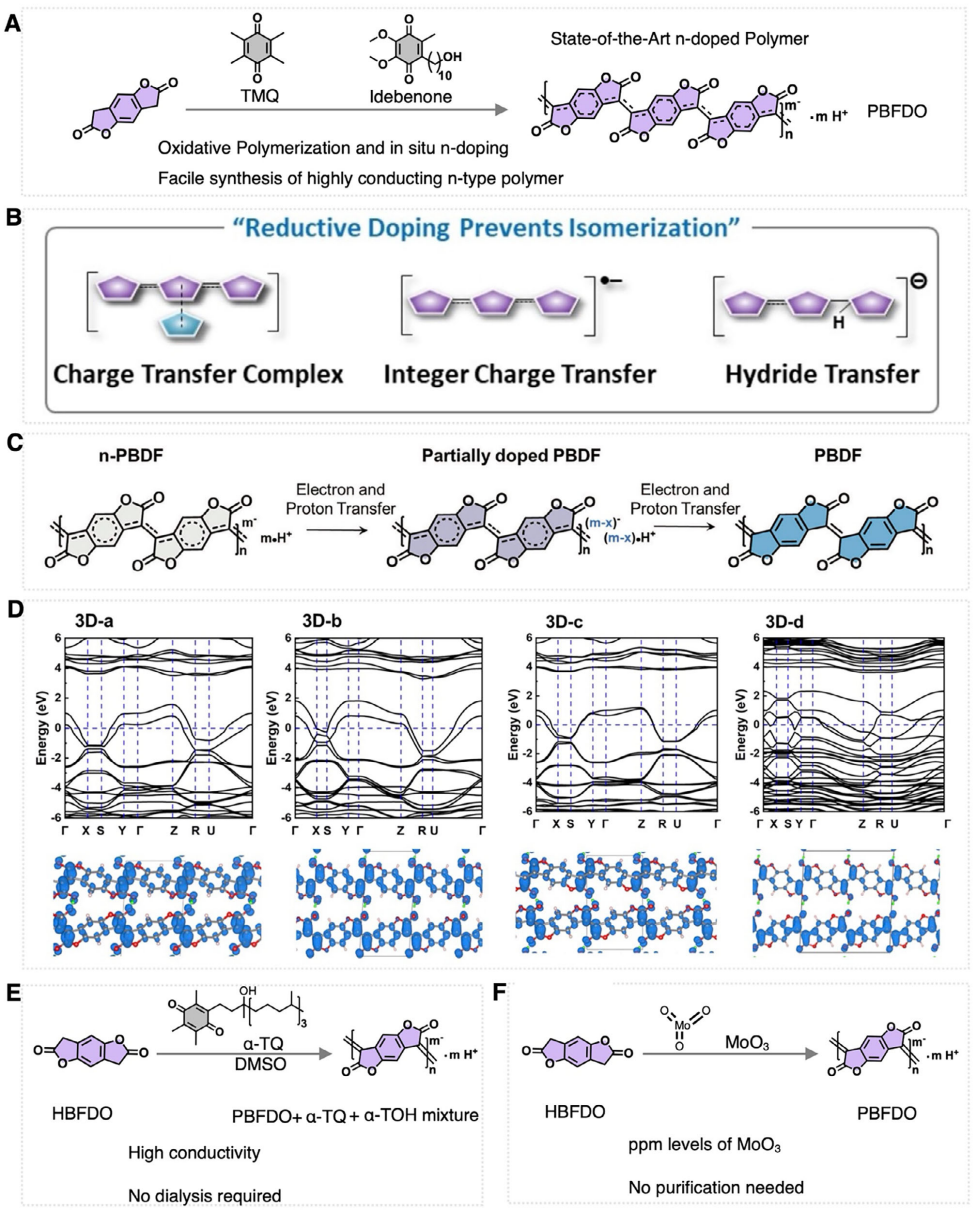
Synthesis and investigations of PBFDO. (A) Schematic diagram of the first synthesis of the state‐of‐the‐art n‐doped polymer PBFDO. (B) Reductive doping prevents the creation of defects due to structural changes (isomerization) in PBFDO. Reproduced under the terms of the CC‐BY Creative Commons Attribution 4.0 International license (https://creativecommons.org/licenses/by/4.0) [96]. Copyright 2024, The Authors, published by Wiley‐VCH. (C) Chemical structures of PBFDO at different doping levels. Adapted under the terms of the CC‐BY Creative Commons Attribution 4.0 International license (https://creativecommons.org/licenses/by/4.0) [97]. Copyright 2024, The Authors, published by Wiley‐VCH. (D). DFT‐PBE0 calculated band structures of the 3D n‐PBDF crystalline networks and partial charge density distributions of the bands crossing the Fermi level. Adapted with permission [99]. Copyright 2024, American Chemical Society. (E) Schematic diagram of PBFDO synthesis using 𝛼‐TQ. (F) Schematic diagram of PBFDO synthesis using MoO_3_.

Among all n‐type π‐conjugated polymers, PBFDO has attracted considerable attention due to its extraordinary electrical conductivity >1000 S cm^−1^ [52, 53]. The polymer was initially reported independently by two research groups in 2022 [52] and 2023 [53], with the synthetic route shown in Figure 3A [52]. Oxidative polymerization and in situ reductive n‐doping operate in combination during the reaction, enabling high doping efficiency. The rigid backbone and the ultra‐low‐lying lowest unoccupied molecular orbital (LUMO) facilitate both efficient electron transport and high delocalization of negative (bi)polarons [52]. Later, convincing evidence emerged that during polymerization, structural isomerization is prevented [96]. Despite high temperatures, structural isomerization can be efficiently inhibited once the hydride transfer adducts, the charge transfer complexes (CTC), or the integer charge transfer (ICT) products are present (Figure 3B), offering a mechanistic explanation for the absence of structural flaws generated from isomerization in the PBFDO backbone [96]. For many practical applications, controlling the doping level of PBFDO is of tremendous importance. Interestingly, controlled dedoping and redoping of PBFDO was reported (Figure 3C) [97], and the process of dedoping involves both electron transfer and proton capture, whereas the dedoped PBFDO can be easily redoped. To provide theoretical support for the high conductivity of PBFDO, the initial first‐principles investigation was based on the one‐dimensional (1D) models, which were insufficient to explain its high n‐type conductivity [98]. Recently, the geometric and electronic features of two‐dimensional (2D) and three‐dimensional (3D) PBFDO networks were investigated using tight‐binding models and first‐principles computations [99]. When examining a coplanar geometry in 2D networks, with BDF moieties confined to protons on the same side, a metallic configuration results. However, backbone torsions break this metallic behavior. In contrast, differences in proton locations and stacking patterns have no effect on the metallic character, which is consistently produced by all 3D designs, as shown in Figure 3D.

The record‐breaking metallic conductivity of PBFDO stems from the absence of solubilizing side chains on the polymer backbone, which, on the other hand, causes a rigid, planar backbone that is brittle and has low mechanical compliance. Consequently, such intrinsic stiffness results in a high Young's modulus and low crack‐onset strain, making PBFDO films difficult to utilize for truly stretchable applications without the use of certain plasticizers or elastomeric blends. Recently, utilizing α‐tocopherylquinone (α‐TQ) as a catalyst (Figure 3E), a new approach for synthesizing PBFDO was demonstrated [54], which removes the necessity for dialysis after the reaction but does not sacrifice the high electrical conductivity, allowing for the production of PBFDO in large quantities. Remarkably, the residual α‐TQ helps overcome the stiffness‐conductivity tradeoff, and by acting as a plasticizer, the modulus dropped by over 10×. Later, a new synthesis method simplified PBFDO production by using DMSO and acetic anhydride (Ac_2_O) as a combined solvent and activator, which triggers the oxidative power of DMSO to catalyze the keto‐enol tautomerism of the BFDO monomer, driving rapid polymerization [100]. The purification‐free, one‐pot synthesis resulted in a high conductivity of 2250 S cm^−1^ in the resulting PBFDO films. Additionally, molybdenum trioxide (MoO3) was used to catalyze the polymerization [101] (Figure 3F). At parts‐per‐million (ppm) concentrations, MoO_3_ achieves a near‐quantitative monomer conversion rate of over 99%, thereby eliminating the necessity for purification. Such a chain‐growth mechanism allows for the synthesis of high‐quality PBFDO with controlled particle sizes and block copolymers. Note that the processing of PBFDO typically requires the use of high‐boiling‐point solvents, such as DMSO, raising concerns regarding high‐temperature thermal annealing as well as solvent toxicity. Recently, utilizing electrostatic interactions between PBFDO and poly(2‐ethyl‐2‐oxazoline) (PEOx), high green solvent (alcohol) processability was achieved in PBFDO: PEOx, enabling both exceptional electrical conductivity beyond 1000 S cm^−^^1^ and remarkable thermal stability at high temperatures up to 250°C [102]. While PEOx does not inherently enhance the absolute conductivity of PBFDO, it functions similarly to how PSS works in the p‐type benchmark PEDOT: PSS. Specifically, the non‐conductive PEOx stabilizes the n‐doped state of PBFDP and offers necessary solubility in green solvent alcohols without losing its metallic‐like conductivity of the resulting films. Notably, such a blending strategy is highly promising for addressing the mechanical flexibility and lays a foundation for next‐generation n‐type stretchable electronics. More recently, the green‐solvent processability of PBFDO was further advanced through the incorporation of a zwitterionic additive, 3‐(N,N‐dimethylmyristylammonio)propanesulfonate (SB3‐14) [103]. The amphiphilic nature of SB3‐14 allows it to interact closely with the PBFDO backbone while providing a hydrophilic exterior. Such dual functionality enables the formation of a stable aqueous ink, thus bridging the gap between metallic‐like conductivity and water‐based processability. Remarkably, SB3‐14 facilitates a lower surface tension and improves the wetting effect of the substrate, allowing for high‐resolution inkjet printing of PBFDO.

In a different approach, Cho et al. employed hydrothermal treatment to induce chemical isomerization of the PBFDO, converting BFDO segments to benzodipyrandione (BPDO) segments [104]. While isomerization reduced carrier density, it yielded superior chain planarity and crystallinity, and more importantly, it created an energy‐filtering effect, resulting in a distinct boost in the Seebeck coefficient up to 48 µV K^−1^ and a high power factor of 142 µW m^−1^ K^−2^. Despite recent breakthroughs in the processing and doping of PBFDO, the synthesis of the polymer still faces significant sustainability hurdles. A major challenge is the continued reliance on DMSO as the essential reaction solvent. The high boiling point of the solvent and the formation of sulfur‐based byproducts during polymerization make it rather difficult for large‐scale production and solvent recovery. Complete elimination of the DMSO phase represents a critical next step in facilitating the large‐scale manufacturing of PBFDO.

#### 2.2.2 N‐type Polymers With Electrical Conductivities Exceeding 100 S cm^−1^

**FIGURE 4:**
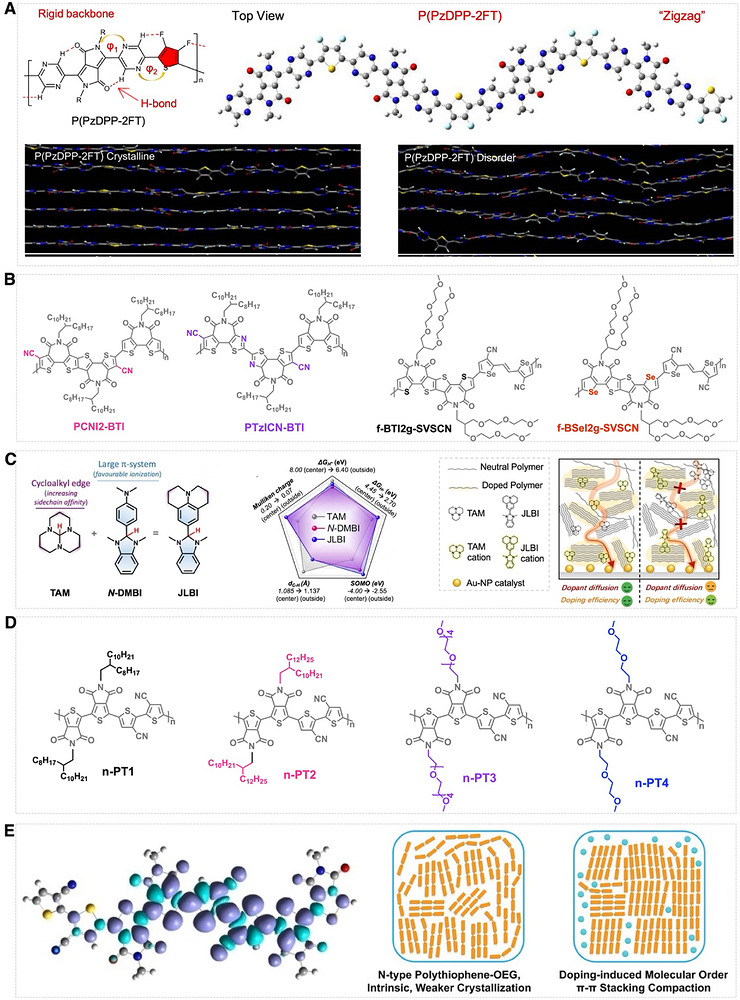
(A) Chemical structure, DFT‐optimized geometry, and MD simulation of P(PzDPP‐2FT). Adapted under the terms of the CC‐BY Creative Commons Attribution 4.0 International license (https://creativecommons.org/licenses/by/4.0) [55]. Copyright 2021, The authors, published by Springer Nature. (B) Chemical structures of PDTzTI‐BTI, PTzICN‐BTI, f‐BTI2g‐SVSCN, and f‐BSeI2g‐SVSCN. (C) Molecular structures of TAM, N‐DMBI and JLBI n‐dopants, radar charm for the theoretical prediction of n‐doping ability for the dopants, and schematic representation of film microstructures f‐BSeIg‐SVSCN after doping with TAM and JLBI. Adapted with permission [60]. Copyright 2024, Wiley‐VCH. (D) Chemical structures of the polymers (n‐PT1, n‐PT2, n‐PT3, and n‐PT4). (E) (left) spin density distribution of the dimer of the n‐PT1 polymer backbone. Adapted with permission [57]. Copyright 2023, Wiley‐VCH. (right) Schematic diagram of doping‐induced molecular order for n‐PT4. Adapted with permission [58]. Copyright 2024, Wiley‐VCH.

During the preceding three to four years, several other n‐type polymers have been reported to exhibit excellent electrical conductivities over 100 S cm^−1^ [105]. The pyrazine (Pz)‐flanked DPP‐based polymer P(PzDPP‐2FT) (Figure 4A) was the first reported solution‐processable polymer giving electrical conductivity exceeding 100 S cm^−1^ [55]. This polymer was designed with the assistance of DFT calculations, which simultaneously considered several characteristics, as shown in Figure 4A. On one hand, the rigid backbone and high‐level coplanarity allow for efficient intra‐and interchain charge transport. On the other hand, the unique zigzag backbone curvature and high/steep torsion‐angle barriers provide robust binding sites for n‐dopants that keep doping levels high but leave polymer packing undisturbed. Together, this strategy can largely reduce the energetic disorder upon doping, achieving an n‐type electrical conductivity in excess of 120 S cm^−1^. BTI and BTI‐analogous units have been recognized as promising building blocks for n‐type thermoelectrics [106]. Recently, fused bithiophene imide dimer (f‐BTI2) and cyano‐functionalized f‐BTI2 (CNI2) were utilized as building blocks in conjugated polymers. Remarkably, the acceptor‐acceptor (A‐A) polymer PCNI2‐BTI (Figure 4B) can be doped to a notably high electrical conductivity of 150.2 S cm^−1^ [59], significantly higher than its analogous D–A polymers. More recently, a novel cyano‐functionalized thienylthiazole imide was designed and copolymerized with a BTI unit, yielding the A‐A polymer PTzICN‐BTI, (Figure 4B), which can be n‐doped to 105.1 S cm^−1^ [107]. Besides, fused bithiophene imide dimer‐based f‐BTI2g‐SVSCN and its corresponding selenophene‐based derivative polymer f‐BSeI2g‐SVSCN were synthesized (Figure 4B, right). As expected, selenophene substitution enables enhanced interchain interactions and a highly ordered molecular arrangement. The polymers were doped with three different n‐dopants, TAM, N‐DMBI, and 9‐(1,3‐dimethyl‐2,3‐dihydro‐1Hbenzoimidazol‐2‐yl)‐julolidine (JLBI), respectively (Figure 4C, left). Hereby, the molecular modification of JLBI involved replacing the dimethylaniline part of N‐DMBI with a julolidine group. This modification increases the lipophilicity of the dopant while keeping its molecular size comparable to that of N‐DMBI. Consequently, JLBI‐doped f‐BSeI2g‐SVSCN benefits from both decent doping efficiency and excellent molecular order (Figure 4C, right), resulting in high n‐doped electrical conductivity exceeding 200 S cm^−1^ and the highest power factor exceeding 100 µWm^−1^K^−2^ [60]. The majority of the high‐performance electron‐transporting conjugated polymers are constructed from building blocks that are composed of fused rings. Interestingly, a non‐fused‐ring approach is demonstrated for designing electron‐accepting polymers, which involves adding electron‐deficient imide or cyano groups to each thiophene unit in a non‐fused‐ring polythiophene backbone, thus leading to an n‐type polymer n‐PT1 with highly delocalized LUMO (Figure 4D,E left) and good electron mobility in solid‐state thin films [108]. When doped with N‐DMBI, n‐PT1displays remarkable electrical conductivity exceeding 60 S cm^−1^ and power factor over 140 µWm^−1^K^−2^. Replacing the side chains of n‐PT1 with bulkier ones and increasing its molecular weight (Mw) yields n‐PT2 (Figure 4D), which can be n‐doped to a higher electrical conductivity of 144 S cm^−1^ but a lower power factor of 75 µWm^−1^K^−2^ [57]. Substituting the alkyl chains with ethylene glycol side chains yields two alcohol‐soluble derivative polymers, n‐PT3 and n‐PT4, and both polymers can withstand high doping loads when n‐doped with n‐DMBI, leading to high electrical conductivity exceeding 100 S cm^−1^ and power factor over 150 µWm^−1^K^−2^ [58]. This excellent performance can be ascribed to their capacity to overcome backbone torsion during doping (Figure 4E, right).

### 2.3 The Development of Ambipolar Thermoelectric Polymers

**FIGURE 5:**
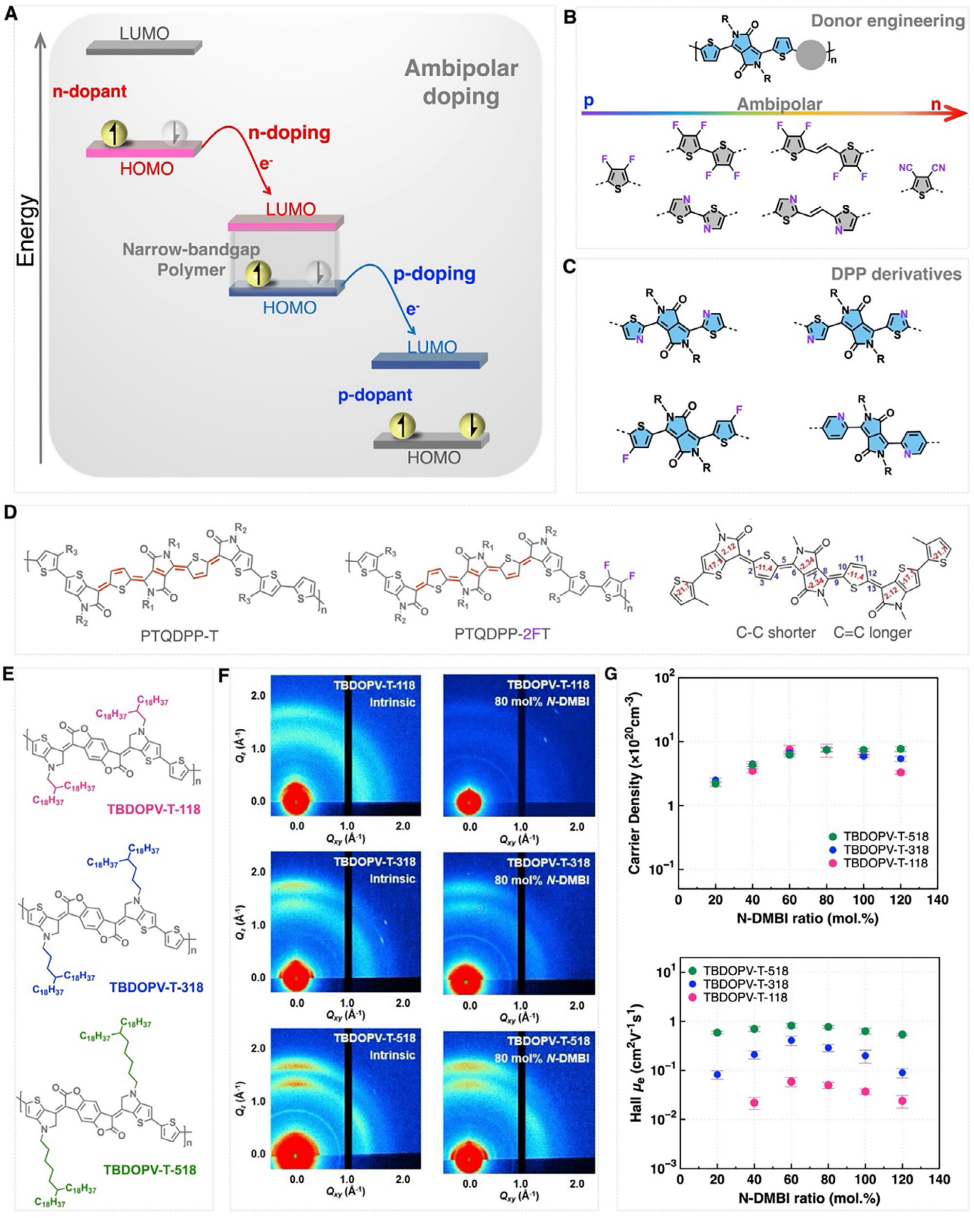
(A) Schematic illustration of ambipolar doping. (B) Donor engineering of DPP‐based polymers for balanced ambipolarity. (C) Chemical structures of DPP derivatives. (D) Chemical structures of PTQDPP‐T, PTQDPP‐2FT, and calculated NICS(1)zz values (in red) of TQDPP. “D, right) Adapted with permission [116]. Copyright 2023, Wiley‐VCH.” (E) Chemical structures of TBDOPV‐T‐118, TBDOPV‐T‐318, and TBDOPV‐T‐518. (F) GIWAXS patterns of pristine and doped films of TBDOPV‐T‐118, TBDOPV‐T‐318, and TBDOPV‐T‐518. (G) Carrier density and Hall mobility as a function of the N‐DMBI molar ratio. “F,G) Adapted with permission [61]. Copyright 2024, American Association for the Advancement of Science”.

It is appealing that a single conjugated polymer can be selectively doped to create both p‐type and n‐type conducting states, as this would enable the fabrication of thermoelectric generators using a single material, thereby significantly reducing manufacturing complexity. Nonetheless, the advancement of ambipolar polymers is far slower than that of their p‐type and n‐type counterparts [109, 110]. This disparity stems from the fact that the design of ambipolar conjugated polymers for thermoelectric applications necessitates a very careful orchestration of electronic structure and backbone conformation. To achieve efficient ambipolar doping, it is essential to precisely align the frontier molecular orbitals to the vacuum level and the respective redox potentials of both types of dopants. More specifically, the HOMO should be sufficiently high to facilitate p‐doping, while the LUMO must be sufficiently deep to enable n‐doping, which naturally necessitates a narrow bandgap (Figure 5A). Consequently, narrow‐bandgap polymers incorporating strong electron‐withdrawing lactam rings, such as DPP‐based polymers [111, 112] and isoindigo (IID)‐based polymers [113, 114, 115], tend to exhibit ambipolarity.

To identify the optimal ambipolar window, rational molecular engineering has been employed to fine‐tune energy levels. For instance, in the case of DPP‐based polymers, introducing electron‐deficient moieties (e.g., F or N) is a widely adopted strategy to achieve balanced ambipolarity. One common strategy involves incorporating these electronegative atoms into the donor unit, which can moderate the electron‐donating character of the donors (Figure 5B). For instance, while the copolymer of DPP and 3,4‐difluorothiophene exhibits predominantly p‐type behavior and slightly n‐type character [117], the addition of an extra 3,4‐difluorothiophene unit promotes quasi‐balanced ambipolarity [118, 119]. Similarly, the copolymers comprising DPP and thiazole‐based units exhibit well‐balanced ambipolarity [120, 121]. Importantly, note that the modulation of the bandgap and frontier energy levels is an exceptionally subtle process, as minor structural modifications may drastically shift the ambipolar window [122]. For instance, the copolymer of DPP and more electron‐deficient 3,4‐dicyanothiophene disrupts the balance, shifting the electronic structure to favor predominantly n‐type behavior in the resulting polymer [123]. An alternative strategy involves functionalizing the DPP core itself with F or N [124, 125], or replacing the thiophene flanking unit with pyridine to yield PyDPP [126], ultimately also leading to satisfactory ambipolarity (Figure 5C). Indeed, ambipolar Seebeck coefficients have been measured in high‐mobility DPP copolymers [127]. Notably, the naphthalenediimide‐diketopyrrolopyrrole polymer (p(gNDI‐TDPP)) can be n‐doped by N‐DMBI and p‐doped by tris(4‐bromophenyl)ammoniumyl hexachloroantimonate [128]. Recently, the incorporation of quinoidal units into the polymer backbones has emerged as a powerful strategy for bandgap compression [129, 130]. The quinoidal structure enforces a rigid, pro‐aromatic backbone that minimizes bond length alternation (BLA) and promotes extended electronic delocalization. Such structural rigidity not only narrows the bandgap but also enhances hole and electron mobility. For example, two DPP‐based polymers, PTQDPP‐T and PTQDPP‐2FT, were synthesized using a quinoidal unit with thienoisatin as the terminus and a thiophene‐flanked DPP unit as the quinoidal core [116] (Figure 5D). Hereby, the C─C and C═C bonds in the TQDPP core are shorter and longer, respectively, than those in the BQDPP analog, resulting in a decreased BLA. Consequently, their high‐lying HOMO and low‐lying LUMO allow for simultaneous p‐doping by FeCl3 and n‐doping by N‐DMBI, leading to asymmetric p‐type and n‐type power factors of >200 µWm^−1^K^−2^ and >2 µWm^−1^K^−2^, respectively [116]. More recently, three benzodifurandione (BFDO) based copolymers (TBDPOV‐T) with different side chains were synthesized. As shown in Figure 5E, three distinct branched side chains were attached to the backbone with the branching points positioned at different distances from the central polymer backbone, yielding TBDOPV‐T‐118, TBDOPV‐T‐318, and TBDOPV‐T‐518, respectively [61]. Among all three polymers, TBDOPV‐T‐518 shows the best tolerance to dopant‐induced disorder (Figure 5F) and exhibits an ideal barrier‐free transport in the doped state. Consequently, under the same doping conditions, the carrier densities are comparable for all three polymers, but the TBDOPV‐T‐518 reaches a significantly higher electrical conductivity (Figure 5G). Finally, when doping TBDOPV‐T‐518 with various dopants, conductivities exceeding 100 S cm^−1^ (for n‐type) and 400 S cm^−1^ (for p‐type), as well as power factors greater than 200 µWm^−1^K^−2^ (for n‐type) and 100 µWm^−1^K^−2^ (for p‐type) were achieved [61]. Besides, a narrow‐bandgap copolymer poly(benzobisthiadiazole‐co‐difluoroquaterthiophene) (PBBT‐4T‐2F) with well‐balanced ambipolar transport feature was doped with the p‐dopant trityl tetrakis(pentafluorophenyl) borate (TrTPFB) and N‐DMBI to achieve selective doping, leading to both p‐type and n‐type behaviors within this single polymer [131]. Taken together, to realize efficient ambipolar doping, the prerequisite is to achieve the optimal ambipolar window by fine‐tuning the energy levels. However, energy level alone does not guarantee high performance. Efficient charge transport in the doped state is also dictated by backbone planarity and intermolecular charge delocalization. Finally, meticulous side‐chain engineering is further required to balance polymer‐dopant miscibility and optimal molecular packing. Future research should focus on polymers that inherently allow for efficient bipolar doping, as this dual functionality guarantees morphological consistency and chemical compatibility across thermoelectric devices.

Table 1 summarizes the thermoelectric performance of state‐of‐the‐art conjugated polymers against their processing conditions and environmental stability. Beyond key thermoelectric metrics, namely σ, S, and PF, the table details the chemical solvents and deposition methods employed to achieve these results. Despite the impressive laboratory breakthroughs highlighted here, the industry‐level adoption of these materials remains in its early stages. A primary concern is that the fundamental ambient and thermal stability of thermoelectric polymers remains largely unexplored, and these challenges deserve more attention and thus will be discussed in detail in Section 6. Furthermore, several critical bottlenecks, specifically regarding production scalability, manufacturing costs, and the reliability of devices during extended operation, remain largely unresolved. These factors, alongside the difficulties of multi‐component electronic integration, are discussed in depth in Section 8.

**TABLE 1:** Thermoelectric performance parameters of state‐of‐the‐art conjugated polymers, including electrical conductivity (σ), Seebeck coefficient (S), and power factor (PF), and beyond performance metrics, alongside key processing solvent, deposition method, and ambient stability.

Polymer	Solvent	Deposition technique	Type p/n	σ (S cm^−1^)	S (µV K^−1^)	PF (µW m^−1^K^−2^)	Film stability in ambient	Refs.
P(DHP‐EDOT)_8_‐*ran*‐(bis‐EDOT)_2_	*o*‐DCB	Blade‐coating	p	1680	—	—	—	[80]
P(DHP‐EDOT)_7_‐*ran*‐(bis‐EDOT)_3_	*o*‐DCB	Blade‐coating	p	1490	—	—	—	[80]
PEDOT:PSS	DMSO	Spin‐coating	p	1864	64.1	765.1	—	[81]
PEDOT:PSS	DMSO	Spin‐coating	p	2254	75	1281	—	[82]
PBTTT‐^8^O	*o*‐DCB	Blade‐coating	p	1100	—	—	—	[85]
pgBTTT	CB	Drop‐casting	p	2180	32	223	—	[91]
PBTTT	*o*‐DCB	Spin‐coating	p	1894	36	176	—	[92]
PIDF‐BTO4	CF	Spin‐coating	p	406	39.7	64	—	[43]
	CF: DCB	Spin‐coating	p	1428	38.3	209.3	—	[43]
	CF: DCB: NMA	Spin‐coating	p	2166	34.9	263.8	—	[43]
PBFDO	DMSO	Drop‐casting	n	∼2000	−21	∼90	95% after 35 days	[52]
	DMSO	Spin‐coating	n	>3000	—	—	>80% after 14 days	[53]
	DMSO	Spin‐coating	n	1321	−30.9	100.4	>97% after 180 days	[54]
	DMSO/Ac2O	Drop‐casting	n	2306	−16	58.7	>87% after 90 days	[100]
	DMSO	Spray‐caoting	n	2247	—	—	>95% after 90 days	[101]
	Water	Spin‐coating	n	∼1400	−36.5	184	ink‐stability in 161 days	[103]
PBFDO: PEOx	Ethanol	Spin‐coating	n	1190	−12	17	ink‐stability in 180 days	[102]
PBPDO	DMSO	Spin‐coating	n	640	−48	142	—	[104]
P(PzDPP‐2FT)	*o*‐DCB	Spin‐coating	n	129	—	—	—	[55]
	*o*‐DCB	Spin‐coating	n	218	—	171	—	[56]
PCNI2‐BTI	CB	Shearing	n	150.2	−118	110.3	—	[59]
PTzICN‐BTI	CF	Spin‐coating	n	105.1	−87.6	50.9	—	[107]
f‐BSeI2g‐SVSCN	HFIP	Spin‐coating	n	206.2	−106.2	114.1	—	[60]
n‐PT2	CN	Spin‐coating	n	144	−72.2	75	—	[57]
n‐PT3	HFIP	Spin‐coating	n	113.8	−105.5	110.0	—	[58]
n‐PT4	HFIP	Spin‐coating	n	133.3	−89.6	100.6	—	[58]
TBDOPV‐T‐518	*o*‐DCB	Spin‐coating	n	114	—	200	—	
	*o*‐DCB	Spin‐coating	p	452	—	>100	—	

**Abbreviations of Solvents**: CB: chlorobenzene; o‐DCB (or DCB): 1,2‐dichlorobenzene; CF: chloroform; CN: Chloronaphthalene; DMSO: dimethyl sulfoxide; HFIP: Hexafluoroisopropanol; NMA: N‐methylaniline.

## 3 Emerging Doping Approaches and Technologies

**FIGURE 6:**
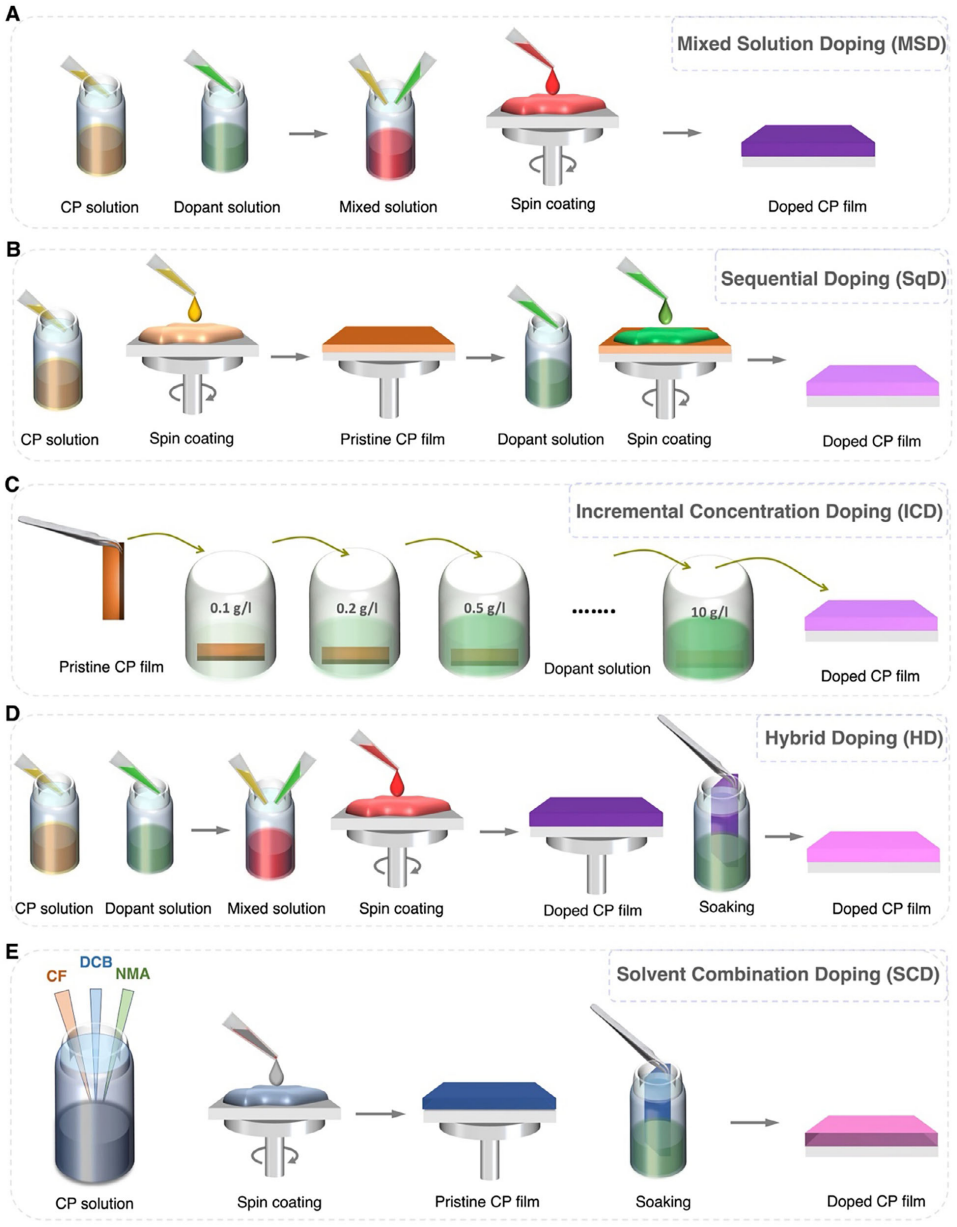
Schematic illustration of solution‐doping of OSCs: (A) Mixed‐solution doping, (B) Sequential doping, (C) Incremental concertation doping, (D) Hybrid doping, and (E) Solvent combination doping.

Doping of semiconducting polymers, originating from the discovery of halogens‐doped, highly conductive “plastic” polyacetylene in the late 1970s by Alan Heeger, Hideki Shirakawa and Alan MacDiarmid [132], has been recognized as an essential enabler in conventional organic electronics devices [48, 133, 134, 135], including organic light emitting diodes [136, 137, 138], organic solar cells [139, 140], organic field‐effect transistors [141, 142], and organic photodetectors [143, 144], thanks to its ability to passivate traps [145], reduce Ohmic losses [146, 147], and enhance operational and ambient stability [148, 149, 150]. In the preceding decade, doping gained even greater popularity and was extensively used to modulate the charge density and optimize the performance of organic thermoelectrics [24, 25, 45]. In earlier times (before 2015), the conventional method (i.e., mixed solution doping) (MSD) has been widely used for doping conjugated polymers (Figure 6A) [151, 152]. However, this approach often suffers from poor polymer‐dopant miscibility and tends to form aggregates even at low doping levels, disrupting the film microstructure and constraining both carrier mobility and electrical conductivity. To overcome this problem, sequential doping (SqD) has been developed, which involves the coating of a dopant solution on top of a pre‐deposited polymer film (Figure 6B) [153, 154]. This approach can effectively reduce the morphological disturbance to the film and result in higher electrical conductivities. Nevertheless, the doping efficiency and stability remain limited. Vapor‐phase doping, which involves exposing a polymer film to the sublimation of dopant vapors [155, 156], is in essence SqD, but it does not always allow for the precise control over the doping level. In recent years, several advanced molecular doping techniques have been developed, and a few unconventional doping strategies have been reported.

### 3.1 Advanced Molecular Doping Techniques

**FIGURE 7:**
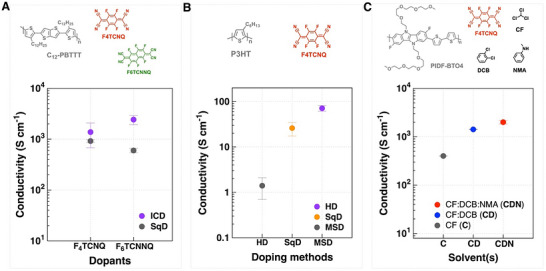
(A) Conductivity as a function of dopants (F4TCNQ and F6TCNNQ) and doping methods (ICD and SqD) for PBTTT‐C_12_. (B) Conductivity as a function of doping methods (HD, SqD and MSD) for P3HT. (C) Conductivity as a function of solvent(s) for PIDF‐BTO4.

To enhance the doping function of classical SqD, a modified form “incremental concentration doping” (ICD), has been developed, which aims for a gradual intercalation of dopant molecules into the polymer films [157]. Specifically, during ICD (Figure 6C), polymer films are doped by consecutively immersing the sample in dopant solutions with progressively higher concentrations. Notably, ICD does not necessarily improve the doping levels as it yields a charge carrier density comparable to that of classical SqD. Rather, it leads to an increase in the charge carrier mobility as a result of preservation of the film morphology as well as the better modified ordering of dopants in the polymer matrix due to the well‐controlled kinetics of dopant diffusion. Consequently, when doping aligned PBTTT‐C_12_, with both F4TCNQ and F6TCNNQ, ICD results in superior electrical performance as compared to that of the classical SqD (Figure 7A) [157]. On the other hand, another novel doping approach has been designed, named hybrid doping (HD), which has the benefit of combining MSD with SqD, thus maximizing the doping efficiency [62]. As illustrated in Figure 6D, the process of HD starts with MSD, and it is followed by sequentially soaking the doped film in a dopant solution, in this way facilitating entire film doping, including both crystalline and amorphous regions, thus resulting in extraordinary electrical performance of the doped films. Taking the doping of P3HT as an example, MSD can achieve a maximum electrical conductivity of 2.1 S cm^−1^, while SqD can produce a conductivity of 34.6 S cm^−1^. After HD, the film exhibits a significant electrical conductivity of 81.5 S cm^−1^ (Figure 7B) [62]. To further enhance the dopant diffusion efficiency in the polymer films, an advanced dopant approach was designed, called solvent combination doping (SCD) [43]. As shown in Figure 6E, this novel method utilizes a combination of solvents in the solution doping process. Hereby, the residual solvents from a ternary solvent mixture increase the free volume in the conjugated polymer film, which enhances the efficiency of dopant diffusion and increases the density of charge carriers. Consequently, the SCD leads to a substantial improvement in the electrical performance for PIDF‐BTO4 (Figure 7C), reaching values up to 2166 S cm^−1^ with the solvent combination of CF, DCB, and NMA. Importantly, this approach can be applied to the efficient doping of a wide variety of conjugated polymers [43].

### 3.2 Emerging Doping Strategies for Polymer Thermoelectrics

**FIGURE 8:**
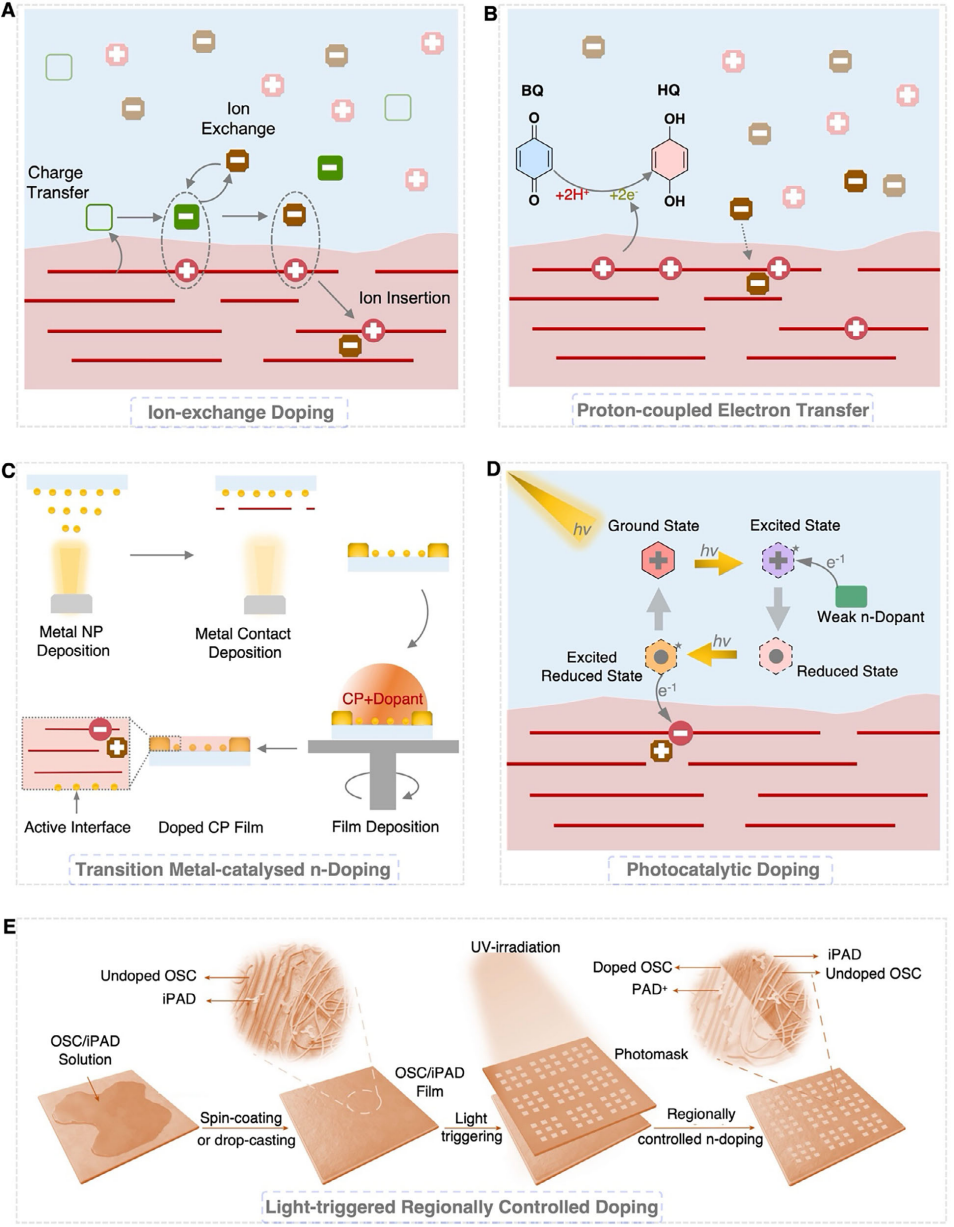
Schematics of the unconventional doping processes. (A) Ion‐exchange doping. Concept based on ref [63]. (B) Proton‐coupled electron transfer. Concept based on ref [165]. (C) Transition metal‐catalyzed n‐doping. Concept based on ref [64]. (D) Photocatalytic doping. Concept based on ref [65]. (E) Light‐triggered regionally controlled doping. Adapted with permission [66]. Copyright 2025, Springer Nature.

**FIGURE 9:**
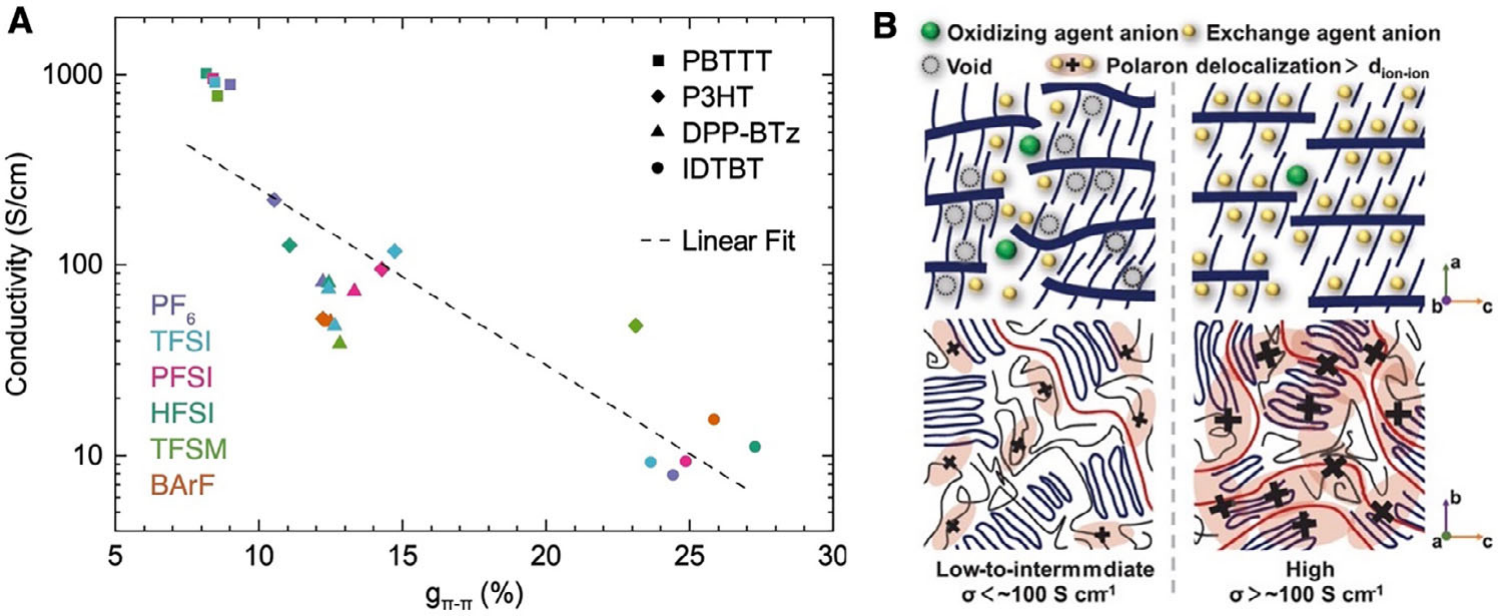
(A) Procrystalline disorder dictates the charge transport in highly doped polymer films. Reproduced under the terms of the CC‐BY Creative Commons Attribution 4.0 International license (https://creativecommons.org/licenses/by/4.0) [159]. Copyright 2022, The authors, published by American Chemical Society. (B) Illustration of film microstructure, dopant habitat, polaron delocalization, and transport pathways in ion exchange doped PBTTT at low‐to‐intermediate and high conductivity regimes. Adapted under the terms of the CC‐BY Creative Commons Attribution 4.0 International license (https://creativecommons.org/licenses/by/4.0) [160]. Copyright 2023, The authors, published by Wiley‐VCH.

Recently, ion‐exchange doping (IExD) was designed and proposed for efficient doping of OSCs [63]. This method is based on exchanging a molecular dopant ion (e.g., F4TCNQ‐), that is bound to a charged polymer with a stable ion (e.g., TFSI‐) from an ionic liquid (Figure 8A). IExD can effectively inhibit coulombic interaction and suppress the formation of ICT, resulting in significantly higher electrical conductivities than those achieved through conventional molecular doping [41, 63, 158]. A further advantage of IExD is that it provides a significantly broader selection of ions sourced from various ionic liquids and salts that are commercially available [41, 63]. It is found that the counterion size had little effect on the electrical conductivity of the highly doped films, indicating that Coulomb traps are not the limiting factors [159]. Rather, the paracrystalline disorder dictates the charge transport and thus the electrical conductivity in highly doped polymer films (Figure 9A). In fact, even at low doping levels, a weak dependence of counterion size is also observed, which can be rationalized by a heterogeneous doping involving the first doping of amorphous domains before proceeding to dope the crystalline domains (Figure 9B) [160]. Recently, a two‐step doping of PBTTT was reported, which combines dopant diffusion in the solid state with subsequent IExD, leading to even higher conductivity than one‐step IExD alone [161]. Initially, most of the reported IExD methods were based on anion‐exchange doping for p‐type OSCs. Very recently, cation‐exchange doping has been developed [162], where the polymer (i.e., the commercially available P(NDI2OD‐T2)) is first reduced by a single electron transfer, followed by the replacement of the ionized dopants with secondary ions through cation exchange [162, 163]. Remarkably, the ambient stability can be improved by choosing proper secondary ions. Similarly, another commercially available polymer, BBL, has also been doped by cation exchange, leading to higher electrical conductivity than conventional doping methods [164].

Proton‐coupled electron transfer (PCET) has been widely utilized in chemistry and biology [166, 167, 168]. Recently, p‐type polymer films were efficiently doped by a two‐electron, two‐proton PCET‐based approach [165]. The process begins with the immersion of polymer films in aqueous solutions containing hydrophobic molecular ions as well as redox pairs (benzoquinone (BQ) and hydroquinone (HQ)) based on PCET (Figure 8B). Then, the effective chemical doping of the polymer films is achieved through synergistic reactions involving PCET and dopant ion intercalation. A strong point of this approach is that BQ and HQ do not react with water, making them highly stable redox agents under ambient circumstances. As per the Nernst equation, simply by tuning the pH of the doping solution, the redox potential of the redox pair and the Fermi level of the polymer semiconductors can be accurately and consistently adjusted [165]. To enhance the n‐doping efficiency of conjugated polymers, a catalyzed n‐doping approach was developed (Figure 8C), in which the cleavage reaction is catalyzed by the incorporation of a transition metal, such as Au, Pt, or Pd, either in the form of vapor‐deposited nanoparticles or in the format of solution‐processed organometallic complexes, such as Pd2(dba)3 [64]. This approach leads to a significant increase in doping efficiency compared to conventional doping methods. Recently, a photocatalytic doping approach was developed based on the use of photocatalysts(PCs) [169, 170], which allows for both p‐doping and n‐doping and the control of doping levels through tuning the dose of light irradiation [65]. In the ground state, weak dopants PCs are not able to dope the polymers; whereas in their excited state, PCs are capable of oxidizing or reducing polymers, and weak dopants are able to regenerate them (Figure 8D). Compared to traditional doping methods that require highly reactive dopants, the photocatalytic doping approach utilizes air‐stable and recyclable PCs, and uses weak dopants like O2 under ambient conditions [65]. Very recently, an advanced light‐triggered doping approach was developed, and a variety of inactive photoactivable dopants (iPADs) were created, which allow for the regionally controlled n‐doping of conjugated polymers [66]. Through transforming iPADs into active dopants using ultraviolet (UV) exposure (Figure 8E), various n‐type polymers can be doped with controllable doping in specific regions, reaching a record resolution as high as 1 µm and allowing for large‐scale fabrication and integration [66]. A few other works that are based on photoinduced doping approaches have been reported. For instance, efficient p‐doping of DPP‐based polymers can be achieved through photoexcitation‐assisted molecular doping, enabling precise patterning of the doped polymers with a resolution up to 1 µm [171]. Notably, the precise doping of conjugated polymers by downscaling the doping resolution to sub‐micrometer scales will enable the future sophisticated flexible electronics [172]. More recently, Cho et al. presented a photoactivated doping approach that facilitates doping‐induced order in solution‐based doping systems, with dopant anions inserted into lamellar regions of the P3HT films, which promotes side‐chain ordering, enhances backbone planarity, and reduces cumulative disorder, leading to high thermoelectric performance [173].

**FIGURE 10:**
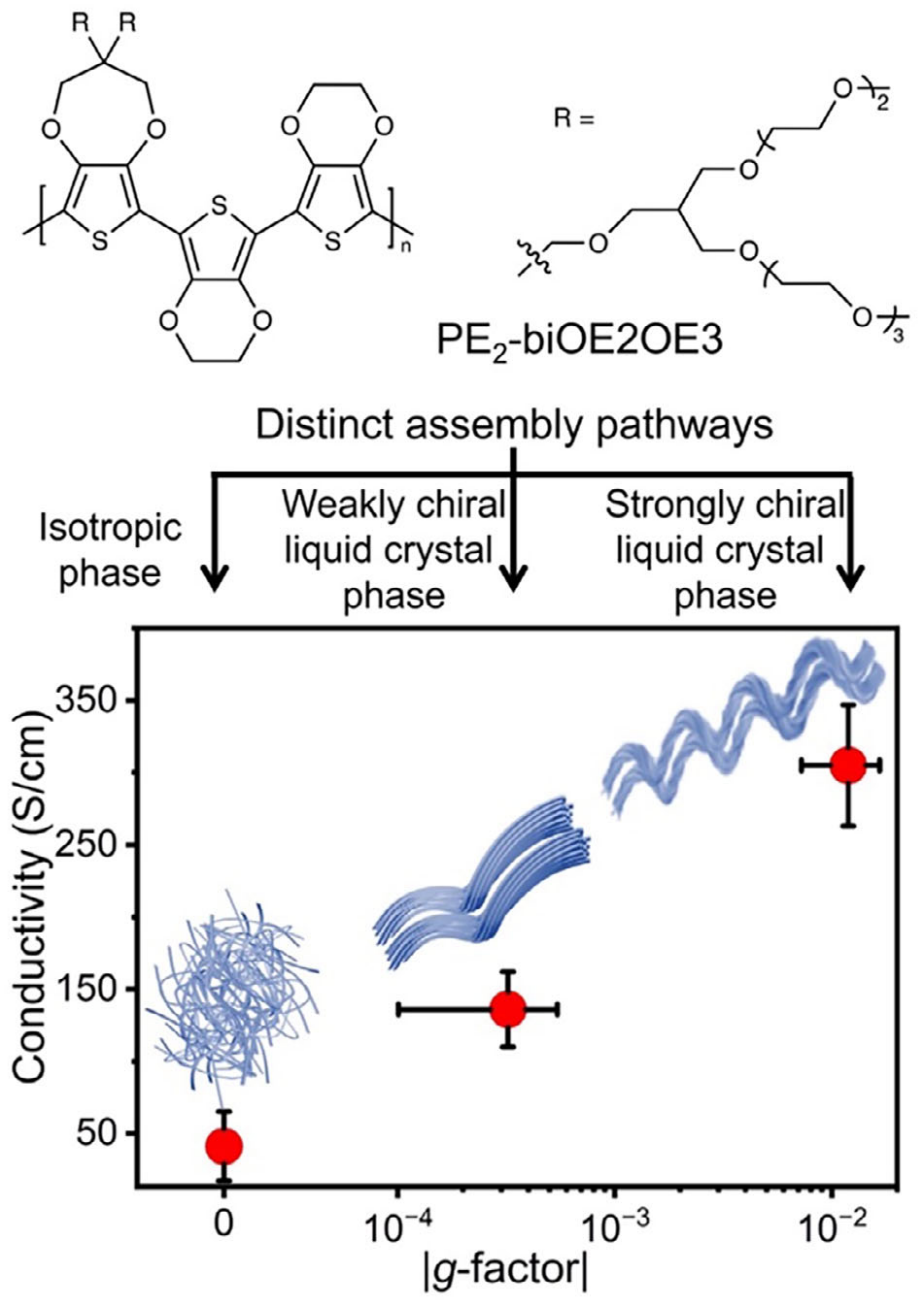
(Top) Chemical structure of PE2‐biOE2OE3 and its solution‐state assembly pathways. (Bottom) Distinct assembly pathways result in films with modulable electrical conductivity upon doping that scales with the absolute dissymmetric factor, |g‐factor |. Reproduced with permission [174]. Copyright 2025, Springer Nature.

Very recently, supramolecular chirality was unexpectedly found to play an essential role in dictating the doping efficiency of conjugated polymers [174]. By controlling the evaporative assembly that occurs during meniscus‐guided coating, chiral helical structures can be formed, with the initially racemic polymer chains first aggregating and then assembling into chiral twist‐bent nematic mesophases. Consequently, the solution‐phase structure is naturally imprinted onto the solid thin film upon drying. Notably, the liquid crystal phase structures can be manipulated by adjusting the aggregate structures of the solution, resulting in a wide range of supramolecular chirality, ranging from achiral to weakly and strongly chiral. Remarkably, the strongly chiral film shows a significantly greater charge carrier density, resulting in the maximum doping efficiency and thus the highest electrical conductivity, whereas the weakly chiral and achiral films perform increasingly worse (Figure 10). The higher doping efficiency can be attributed to: first, the enhanced crystallinity from the chiral assembly makes the doping easier; and second, chirality‐induded spin selectivity speeds up the oxidation reaction more quickly [174]. This revolutionary observation has opened a fresh avenue for leveraging chirality to modulate chemical doping. With the development of chiral material design [175], this strategy holds huge potential in the future modulated doping of conjugated polymers for various applications.

## 4 Emerging Strategies for Suppressing Dopant‐Induced Disorder

In addition to designing disorder‐tolerant conjugated polymers [55], various strategies have been developed to suppress dopant‐induced disorder for enhancing the thermoelectric performance.

### 4.1 Random Copolymerization for Suppressing Dopant‐Induced Disorder

**FIGURE 11:**
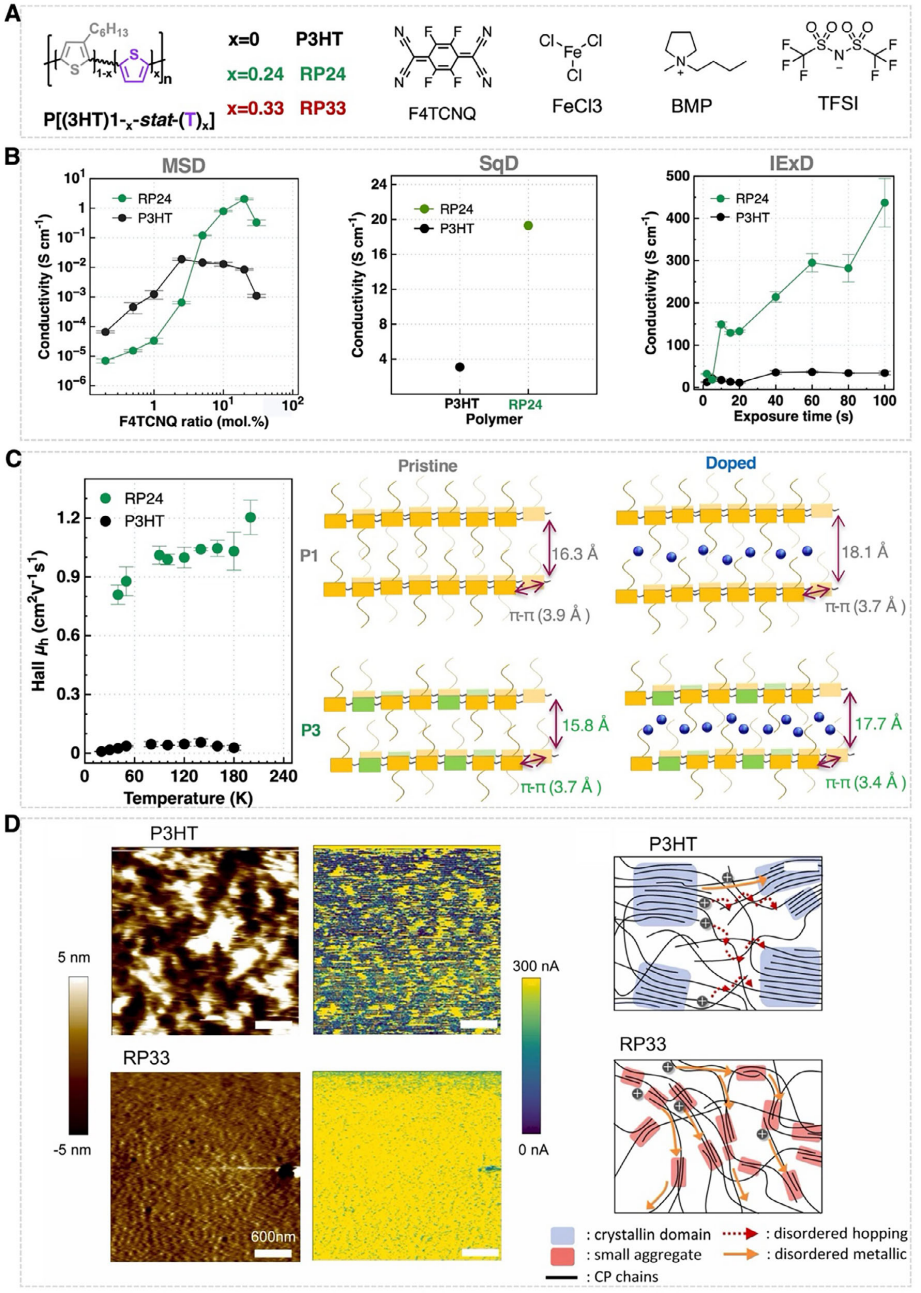
(A) Chemical structures of P[(3HT)_1‐x_‐*stat*‐(T)_x_] and corresponding dopants. (B) Electrical conductivities of P3HT and RP24 doped by different doping approaches. (C) Hall mobilities of P3HT and RP24 doped by IExD and schematic illustrations of molecular packing of the two polymers before and after doping. (D) c‐AFM images (left) and schematic illustrations of heterogeneous transport in P3HT and RP33 (right). “B) (left) Adapted with permission [184]. Copyright 2023, The authors, published by Elsevier. B) (middle) Data reported by ref [185]. B) (right) and C) (left) Adapted under the terms of the CC‐BY Creative Commons Attribution 4.0 International license (https://creativecommons.org/licenses/by/4.0). Copyright 2024, The authors, published by Wiley‐VCH [70]. D) Adapted with permission [183]. Copyright 2025, Elsevier”.

Random copolymerization enables tuning the side chain density/category and the simultaneous adjustment of the long‐range/short‐range order of the conjugated polymers [176, 177]. Previously, random copolymerization of 1D and 2D monomers (i.e., DPP‐EDOT and BDTTT‐DPP) alleviated the trade‐off between the Seebeck coefficient and electrical conductivity, leading to higher thermoelectric performance [178]. Moreover, tuning the ratio of DPP‐3T and EDOT in their random copolymers significantly enhanced their thermoelectric performance [177]. Similarly, the thermoelectric performance of random copolymers increased with the ratio of oligo(ethylene glycol)‐substituted bithiophene g_3_2T‐TT over DPP‐TT up to an optimal point, after which it began to decline [176]. The enhancement is attributed to the addition of g_3_2T‐TT, which raised the HOMO level of the polymer for improved charge transport and increased dopant‐polymer miscibility due to its polar side chains. Notably, random copolymerization of 3‐hexylthiophene (3HT) and unsubstituted thiophene (T) units yields the copolymer P[(3HT)_1‐x_‐*stat*‐(T)_x_], where the steric hindrance brought by the side chains can be adjusted by altering the ratio of the T unit (Figure 11A). Adding a consecutive pendant‐free T moieties lessens the steric hindrance and simultaneously enhances the aggregation extent in comparison to the homopolymer [179, 180]. As a result, the poorly ordered microstructure (with reduced long‐range order) of random copolymers in the solid state does not necessarily limit their charge carrier mobility, provided that short‐range order (i.e., local aggregations) is maintained [181, 182]. Upon doping, the presence of weakly ordered, local aggregates may sustain the pathway for efficient charge transport [181]. Meanwhile, the molecular cavities in random copolymers facilitate the loading of dopant counterions without significantly disrupting the microstructure.

Notably, the random copolymer containing 24% T units (RP24, Figure 11A) can be doped to much higher electrical conductivities regardless of dopant (Figure 11A) and doping approach, whether it is MSD [184], SqD [185] or IExD [70] (Figure 11B). Notably, the random copolymer RP24 exhibits comparable charge carrier mobility to the homopolymer P3HT in the pristine state (though marginally higher for RP24), independent of the device structure, whether it is in bottom‐gate [184, 185] or top‐gate transistors [70]. Remarkably, after doping(e.g. IExD), the Hall mobility of the doped P3 significantly outperforms that of the doped P3HT (Figure 11C), which can be explained by the highly suppressed disorder in the doped RP24, that the doping promotes the planarization of the polymer backbone, shortens the 𝜋–𝜋 distances (Figure 11C) [186], facilitating greater molecular orbital overlap for a more efficient charge transport, leading to an impressive electrical conductivity of the doped P3 films (> 400 S cm^−1^) [70]. A very recent study by Cho et al. revealed that the 𝜋–𝜋 aggregates in the random RP33 act as effective transport junctions to establish continuous percolation pathways, which promote charge delocalization and inhibit charge trapping, thus resulting in a change from hopping‐based to metallic‐like conduction [183] (Figure 11D, left). Indeed, this insight was visualized in conductive‐AFM images (Figure 11D, right), where P3HT showed a strong current disparity between domains and domain borders with relatively low current, whereas the random RP33 showed little variation but with significantly greater current levels.

### 4.2 Controlling Dopant Insertion: Modulation Doping and Counterion Docking

**FIGURE 12:**
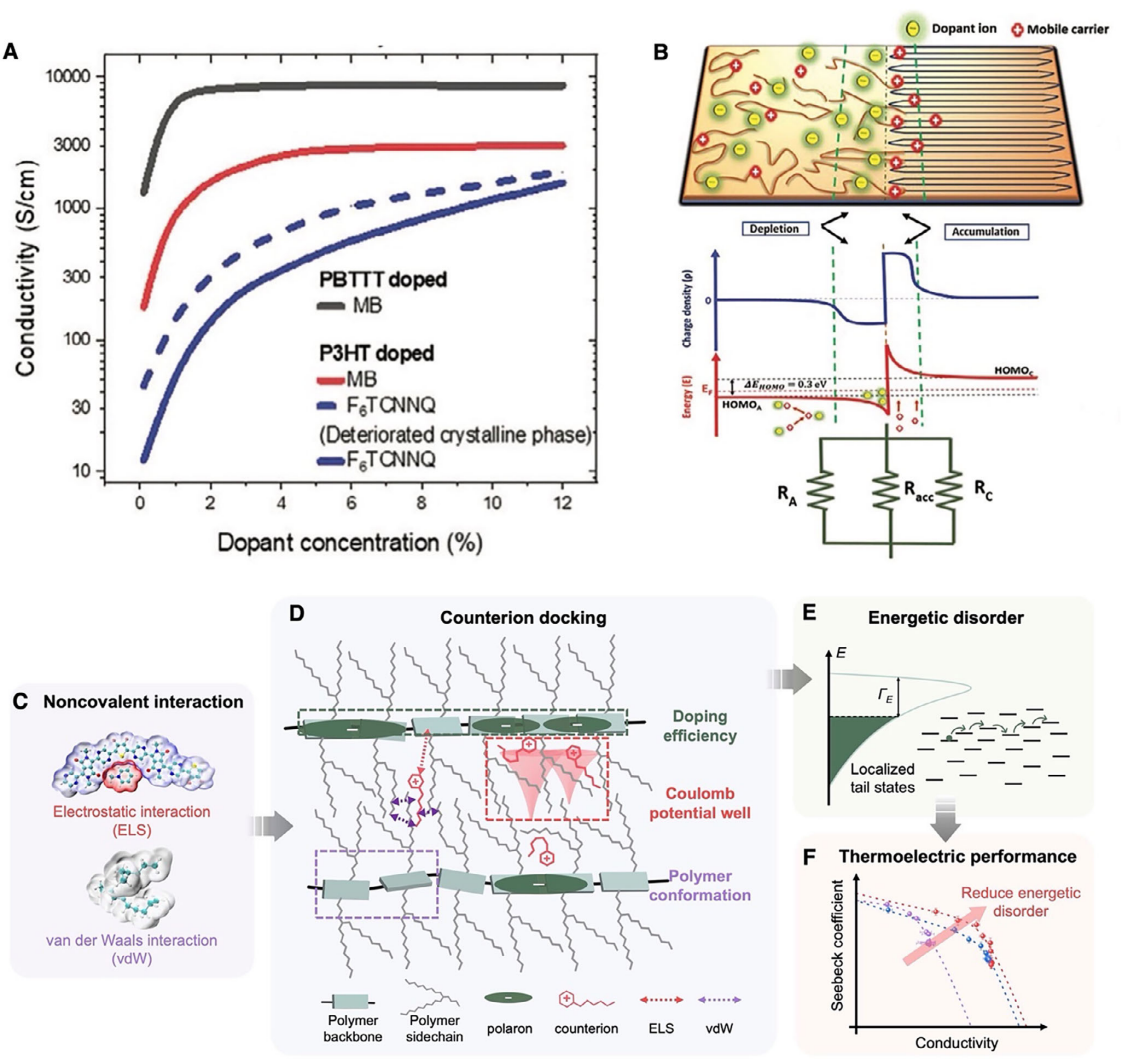
(A) Spontaneous modulation doping with MB leads to a rapid rise in electrical conductivity to extraordinarily high values, and followed by saturation. (B) The model illustrates the modulation doping. (C–F) Schematic illustration of how the noncovalent interactions (NCIs) affect the charge transport properties and thermoelectric performance. “A,B) Adapted under the terms of the CC‐BY Creative Commons Attribution 4.0 International license (https://creativecommons.org/licenses/by/4.0) [42]. Copyright 2024, The Authors, published by Wiley‐VCH. C–F) Adapted under the terms of the CC‐BY Creative Commons Attribution 4.0 International license (https://creativecommons.org/licenses/by/4.0) [56]. Copyright 2024, The Authors, published by Springer Nature”.

In the field of inorganic semiconductors, dopant insertion can deteriorate the quality of the crystal structure, increase impurity scattering, and thus reduce the charge carrier mobility; such negative effects can be lessened through a technique called modulation doping, which was initially proposed by Dingle et al. for group III‐V semiconductors [187], and later adapted by König et al. for silicon [188]. Modulation doping involves contacting a narrow bandgap semiconductor with a substantially doped broad‐bandgap semiconductor [189], and this allows for spatially separating conduction electrons from their parent donor impurity atoms, thus mitigating the impact of neutral and ionized impurity scattering on electron mobility [187]. Notably, modulation doping was successfully applied in conjunction with high‐mobility crystalline rubrene thin films, enabling the achievement of higher doping efficiencies and greater thermoelectric power factors than those of conventional bulk doping [190]. However, the modulation doping for the polymer systems has been rarely reported. Recently, the first report on the modulation doping of conjugated polymers suggested that appropriately engineered semi‐crystalline polymer films can undergo spontaneous or self‐organized modulation doping as long as two factors are fulfilled: i) the polymer morphology consists of crystalline and amorphous phases, with the latter preferably connected over long ranges, and ii) the dopant dwells predominantly in the amorphous phase [42]. These two requirements, when met, result in a very efficient doping even when the dopant load is low, which is characterized by a quick rise in electrical conductivity to extraordinarily high values before saturation (Figure 12A). A model was established to describe the modulation doping of the polymers, (Figure 12B), that dopants only reside in the amorphous region and the band offset, which is formed by the distinct microstructures of the two phases, facilitates the migration of the mobile charges to the crystalline phase, such that an ion‐pure depletion zone forms in the amorphous phase, whereas a carrier‐only accumulation regions forms at the the interface with crystalline‐phase side. Both semi‐crystalline P3HT and PBTTT show spontaneous modulation doping when doped with magic blue (MB). Nonetheless, the majority of newly developed high‐performance polymers are low‐crystallinity D–A copolymers with a limited crystalline area. Thus, it remains an open question whether spontaneous modulation doping may be applied to a wider range of polymeric systems.

Unlike the doping of inorganic semiconductors, where covalent bonds are the major force, the doping of conjugated polymers involves intermolecular non‐covalent interactions (NCIs) between the dopants and polymers (Figure 12C), and molecular packing (in other words, dopant‐induced disorder) depends critically on these NCIs [191]. However, systematic investigations of how NCIs affect doping are rarely reported. Recently, a computer‐assisted approach was proposed for screening a wide range of counterions in an effort to identify the most effective one for efficient and rational doping [56]. Notably, the NCIs dictate the counterions' distribution and docking sites, which in turn impact the Coulomb potential landscape, doping efficiency, polymer backbone planarity, as well as energetic disorder (Figure 12D,E). Together, these NCIs determine the final thermoelectric power factor (Figure 12F). Remarkably, utilizing this strategy, dopant‐induced disorder can be significantly reduced, and extraordinarily high electrical conductivity (> 200 S cm^−1^) can be achieved, ultimately leading to an improvement in power factor by eight‐fold [56].

### 4.3 Side‐Chain Cleavage of Conjugated Polymers for Reduced Disorder

**FIGURE 13:**
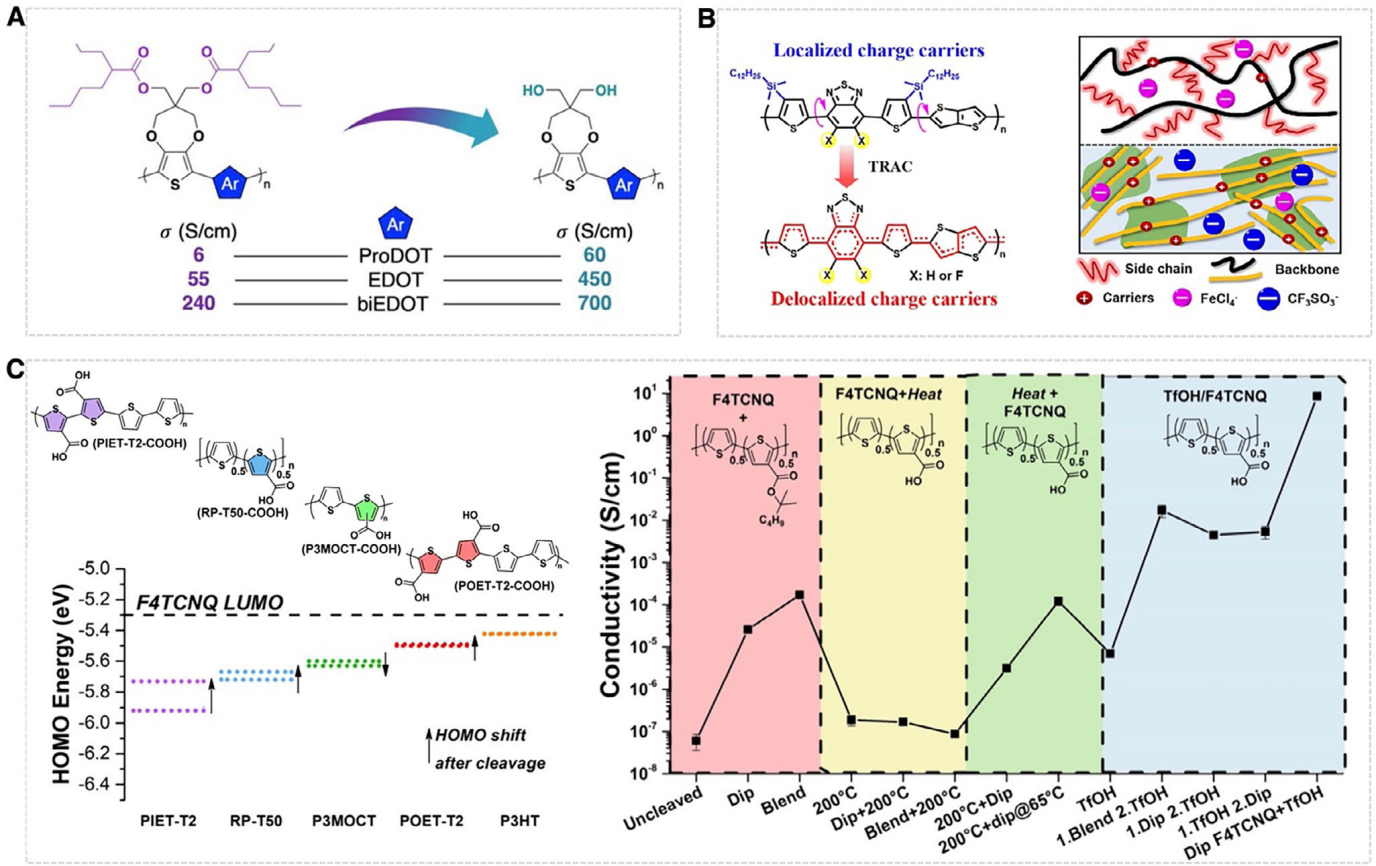
(A) Side chain removal of ProDOT‐based copolymers via ester hydrolysis. Reproduced with permission [67]. Copyright 2022, American Chemical Society. (B) Side‐chain‐free BT‐based conductive polymers by thermal‐assisted rapid acid cleavage. Adapted with permission [69]. Copyright 2025, Wiley‐VCH. (C) Acid‐triggered side chain cleavage of polythiophene‐carboxylic acid‐based polymers(left) and the advantage of “cleavage with doping” over conventional doping methods(right). Adapted with permission [196]. Copyright 2025, American Chemical Society.

Side chains are responsible for the solubility and processability of conjugated polymers; as such, they are of vital importance in modulating the self‐assembly characteristics of the polymers [192]. On the other hand, the presence of these insulating side chains lowers the proportion of the electroactive material (i.e., the conjugated backbone) in the solid‐state thin films. This can hinder molecular orbital overlap, enlarge 𝜋–𝜋 intermolecular distances, localize the charge carriers, and sacrifice achievable optoelectronic properties. Indeed, when the side chain length is decreased in poly(3‐alkylthiophene)s (P3ATs), the interchain π−π stacking distance of the doped thin films becomes shorter, and the electrical conductivity gradually increases [193]. To better address the side‐chain‐induced problems, several post‐treatment methods have been developed to remove the side chains after solution processing, often leading to intensely packed films, thus eliminating the deteriorating effect of the side chains. One effective strategy is ester hydrolysis [194], which was recently employed to remove the side chains of 3,4‐propylenedioxy‐thiophene (ProDOT)‐based copolymers consisting of a ProDOT core functionalized with 2‐butyloctyl ester (BOE) that is a copolymer unit (Figure 13A) [67]. Remarkably, by eliminating the insulating side chains, the resulting solvent‐resistant films can be doped to significantly higher electrical conductivity values under the same doping conditions, ca. 1 order of magnitude higher. Another commonly used approach is the acid cleavage of silyl‐based side chains [195]. For instance, side‐chain‐free benzothiadiazole (BT)‐based conductive polymers were produced utilizing a thermal‐assisted rapid acid cleavage(TRAC) (Figure 13B, left) [69]. This approach enables the complete elimination of silane side chains from the original polymer and the initial doping with trifluoromethanesulfonic acid (TfOH). Notably, the Hall mobility of the side‐chain‐free polymers significantly outperforms that of the parent polymers, due primarily to the enhanced backbone packing of the backbone following the removal of the side chains (Figure 13B, right). Notably, an outstanding electrical conductivity of 730 S cm^−1^ and a power factor of 81 µWm^−1^K^−2^ are achieved, ranking it the highest for BT‐based polymers. In another work, thiophene‐based polymers with tertiary ester side chains were synthesized, where the side chain cleavage resulted in a shift in their HOMO levels (Figure 13C, left). [196] By combining the p‐dopant F4TCNQ and trifluoromethanesulfonic (TfOH) as a strong acid, a “cleavage with doping” approach was designed (Figure 13C, right), which simultaneously removed the side chains and doped the polymers, resulting in a remarkable electrical conductivity > 350 S cm^−1^. In a similar fashion, a range of benzodithiophene (BDTTT) copolymers (PBDTTT‐TET_x_) were synthesized featuring ester‐cleavage groups which are easily broken down by the widely employed FeCl_3_, allowing for the simultaneous side‐chain removal and chemical doping, leading to an impressive 120‐fold increase in electrical conductivity and a 15‐fold rise in power factor [68]. In general, side‐chain cleavage is an effective strategy to suppress disorder and enhance the thermoelectric metrics of conjugated polymers.

## 5 Unconventional Strategies to Overcome the Trade‐Off in Polymer Thermoelectrics

**FIGURE 14:**
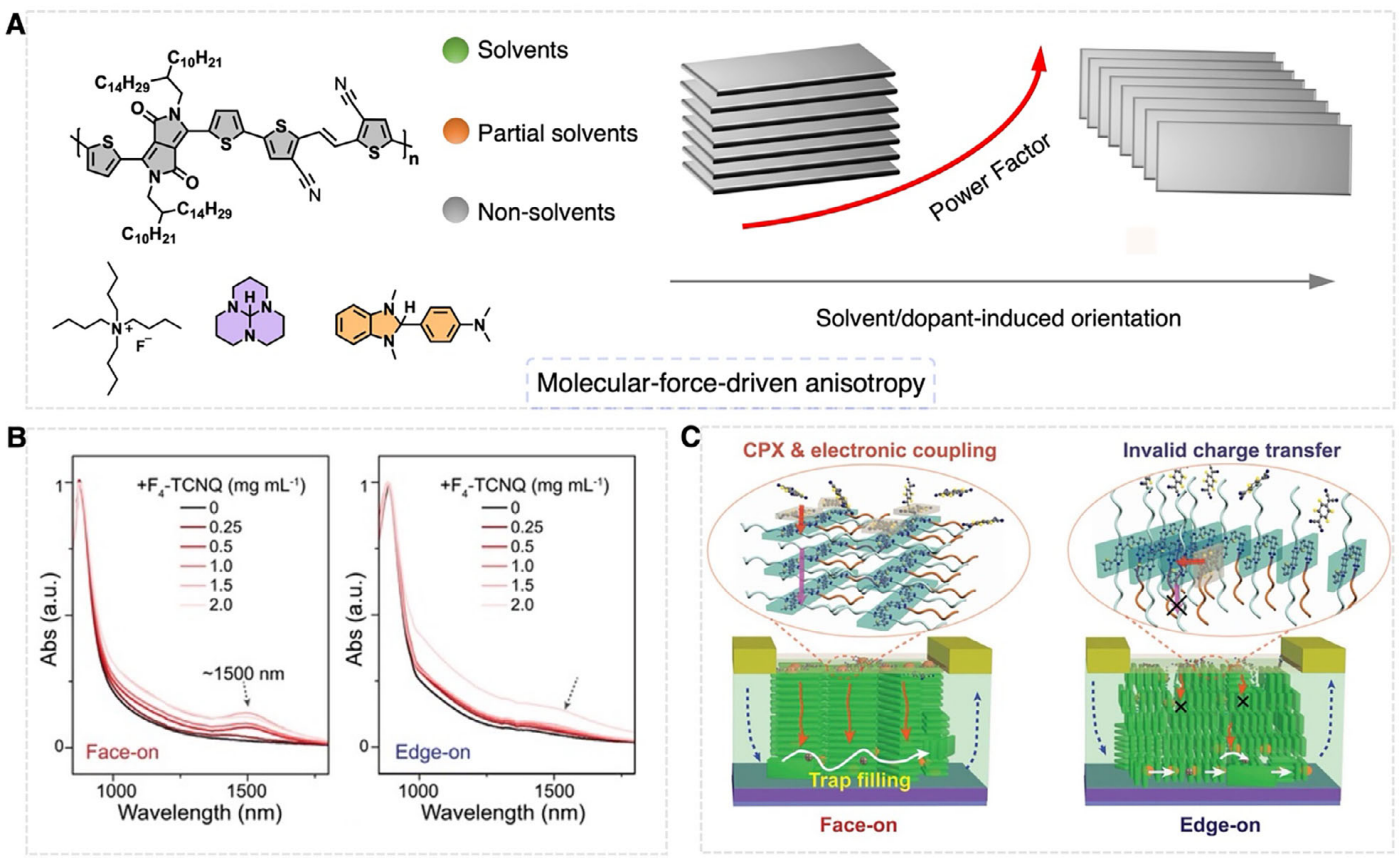
(A) Molecular‐force‐driven anisotropy allows for decoupling the parameters in polymer thermoelectrics. Concept based on ref [72]. Copyright 2025, Springer Nature. (B) UV–Vis–NIR absorption spectra for DPP‐BTz films of face‐on and edge‐on orientation, surface doped with F4‐TCNQ at different concentrations. (C) Schematic illustration for the charge transfer and transport processes in the surface doped DPP‐BTz films comparing face‐on and edge‐on orientations. “B,C) Adapted with permission [207]. Copyright 2020, Wiley‐VCH”.

Conventionally, three primary strategies are employed to decouple the electrical conductivity and Seebeck coefficient: i) DOS engineering, which enables increasing the Seebeck coefficient without compromising electrical conductivity [197, 198]; ii) energy filtering, which can significantly enhance the Seebeck coefficient [73, 199, 200]; and iii) polymer chain alignment, which can increase charge carrier mobility and electrical conductivity while keeping the Seebeck coefficient invariant [201, 202, 203]. In order to induce morphological anisotropy, additional mechanical treatments are typically required, such as high‐temperature rubbing [203, 204], and various meniscus‐guided aligning techniques [205, 206]. To bypass the need for mechanical treatment, a molecular‐force‐driven anisotropy (MFDA) approach was designed, which yields polymer films with a preferred orientation by initiating a self‐assembly process modulated by forces at the molecular level that act between conjugated polymers, solvents, and dopants (Figure 14A). More specifically, this method is based on intermolecular interactions, which are assessed using the Hansen solubility parameters framework, to offer predictive criteria for solvent selection of semiconducting polymers that yield films with a preferential molecular orientation [72]. According to kinetic Monte Carlo simulations, it is predicted that edge‐on orientations of the semicrystalline polymers lengthen the in‐plane delocalization length, thereby overcoming the conventional trade‐off between the electrical conductivity and the Seebeck coefficient. These guidelines are noteworthy because they are applicable to both the copolymer 2DPP‐2CNTVT (Figure 14A) and the homopolymer P3HT [72]. It is worth mentioning that an edge‐on orientation may not necessarily be the most favorable scenario for efficient thermoelectric performance. For instance, it was observed that films with an edge‐on orientation exhibited higher carrier mobility before doping, whereas films with a face‐on orientation demonstrated superior performance after doping [207]. As shown in the UV–vis spectra (Figure 14B), the intensity of the charge‐transfer complex absorption peak is much stronger in the face‐on orientation, suggesting that this packing mode is more favorable for doping. Indeed, as illustrated in Figure 14C, the face‐on alignment effectively exposes the conductive backbone's reaction sites to the dopant molecules, whereas edge‐on orientation tends to impede the interaction between dopants and the backbone due to the presence of the insulating alkyl side chains. In fact, in a few recent studies, the coexistence of both face‐on and edge‐on mixed orientations of the doped films was observed to outperform those based on mono‐type orientations [208, 209]. Regardless of the optimal packing modes, MFDA allows for the fine‐tuning of the orientation of conjugated polymers. Nevertheless, limitations may exist that MFDA applies only to materials with significant crystallinity, whereas it does not take into consideration charge transport in amorphous regions. To answer these questions, further investigations are required in the future.

**FIGURE 15:**
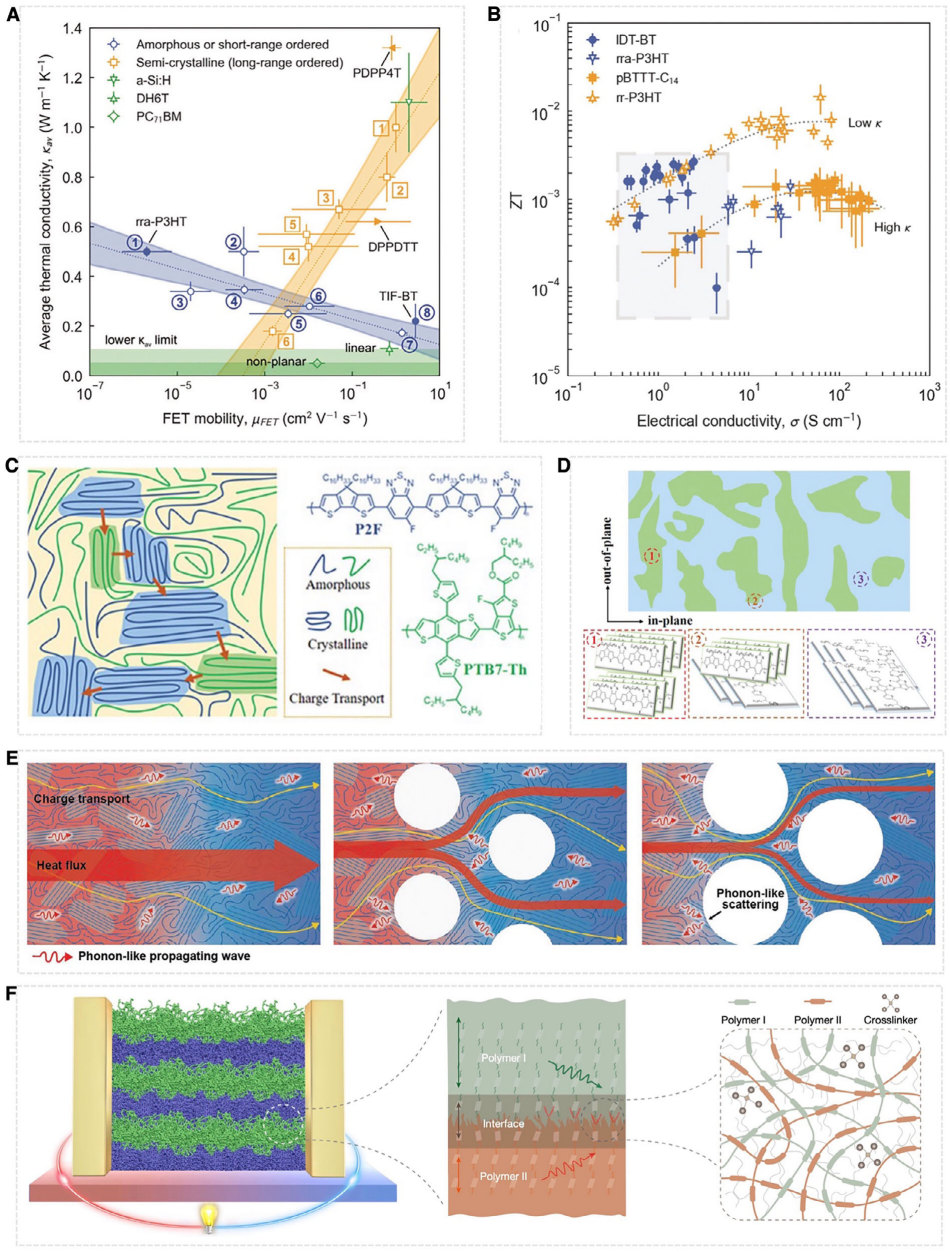
Emerging strategies decouple the thermal and electronic transport in polymer thermoelectrics. (A) κ of conjugated polymers and conjugated molecules vs. field‐effect mobility. (B) Figure‐of‐merit ZT of archetypal conjugated polymer films. “A,B) Adapted under the terms of the CC‐BY Creative Commons Attribution 4.0 International license (https://creativecommons.org/licenses/by/4.0) [12]. Copyright 2024, The Authors, published by Wiley‐VCH”. (C) Illustrative polymer blend morphologies between high‐𝜎 polymer P2F and high‐S polymer PTB7‐Th and their corresponding molecular structures. (D) Schematic illustration of the blend film morphologies in the out‐of‐plane direction. “C,D) Adapted with permission [211]. Copyright 2024, Wiley‐VCH”. (E) Schematic diagram of charge and heat transport in the pristine and porous films. Adapted with permission [212]. Copyright 2024, Wiley‐VCH. (F) Multi‐heterojunctioned plastics. Adapted with permission [71]. Copyright 2024, Springer Nature.

Conventionally, the understanding of the critical parameter thermal conductivity (κ) in organic thermoelectrics is fragmented, due primarily to the difficulties in the precise and extensive characterizations of conjugated polymers. Recently, Campoy‐Quiles et al. statistically measured κ across a variety of conjugated molecules, ranging from long‐range ordered polymers (such as rr‐P3HT, PBTTT) to near‐amorphous polymers (such as IDT‐BT, rra‐P3HT) and observed two notably distinct thermal transport regimes that are dictated by microstructure [12]. Specifically, in the former case, thermal transport adheres to classic theories that thermal transport and charge transport are coupled, leading to simultaneously higher charge carrier mobility and higher κ with increased long‐range ordering (Figure 15A). In sharp contrast, in the latter scenario, the electronic transport and thermal transport are decoupled, so that κ decreases even as charge mobility increases, resulting in a 10‐fold improvement in the ZT in the quasi‐amorphous polymer IDT‐BT over its semi‐crystalline counterpart (i.e., PBTTT) when both polymers are comparable in their electrical conductivities (Figure 15B). More recently, the same research group observed that the microstructure of conjugated polymers can be altered upon doping, leading to a change in κ of the polymer films [210]. Again, opposite trends in the impact of doping on κ were revealed for polymers with different crystallinity. In highly ordered polymers, doping decreased the out‐of‐plane κ; whereas in less ordered polymers, doping increased the out‐of‐plane κ. Finally, in‐plane alignment of the polymer chains was held accountable for the impact on κ in all directions, thus underscoring the importance of measuring both electrical and thermal properties in the same orientation, either both in‐plane or both out‐of‐plane; failing to do so will lead to inaccurate reports of ZT values [210].

To decouple the thermal and electrical transport properties, a ′film sequence′ strategy was employed, which involved blending two different polymers, one with high electrical conductivity (P2F) and another with high Seebeck coefficient (PTB7‐Th) [211] (Figure 15C). In this way, 3D Conductive paths were formed at the composition of 35%–80%, leading to enhanced charge transport. The maximum power factor was achieved at 65% PTB7‐Th, which can be attributed to the simultaneous presence of both bilayered and mixed morphologies in the blend film (Figure 15D). As the amount of PTB7‐Th increases, the bilayered morphology ensures that the electrical conductivity does not drop substantially, whereas the mixed morphology guarantees the enhancement of the Seebeck coefficient [211]. Note that the blend film exhibits notably lower thermal conductivity than each of the polymers, resulting in a high ZT value of 0.15. Remarkably, Di et al. employed imprinted porous polymer films to scatter and impede phonon transport while preserving pathways for charges [212] (Figure 15E). Such a design strategy was able to slash κ by 50%, resulting in a boost in ZT by 170% to a peak value of 0.52. Recently, high‐ZT thermoelectric plastics were obtained by the same group of researchers via developing a multi‐heterojunction with periodic dual‐heterojunction characteristics [71] (Figure 15F). Here, each period consists of two polymers arranged in a heterojunction interface thicker than 10 nm. Such a geometry results in a substantial increase of interfacial phonon‐like scattering while still allowing for effective charge transport, leading to a considerable reduction in thermal conductivity and substantial improvement in power factors, achieving a quasi‐“phonon‐glass electron‐crystal” state. Finally, at 368 K, the thermoelectric plastics exhibited a ZT value of 1.28, a record for polymer thermoelectrics [71]. Importantly, this strategy is compatible with solution processing techniques over large areas, holding high potential for practical applications.

## 6 Air and Thermal Stability of Polymer Thermoelectrics

A major obstacle to the commercialization of organic thermoelectrics is their stability issues, including air stability (i.e., dedoping issue) and thermal stability.

### 6.1 Air Stability of Polymer Thermoelectrics

**FIGURE 16:**
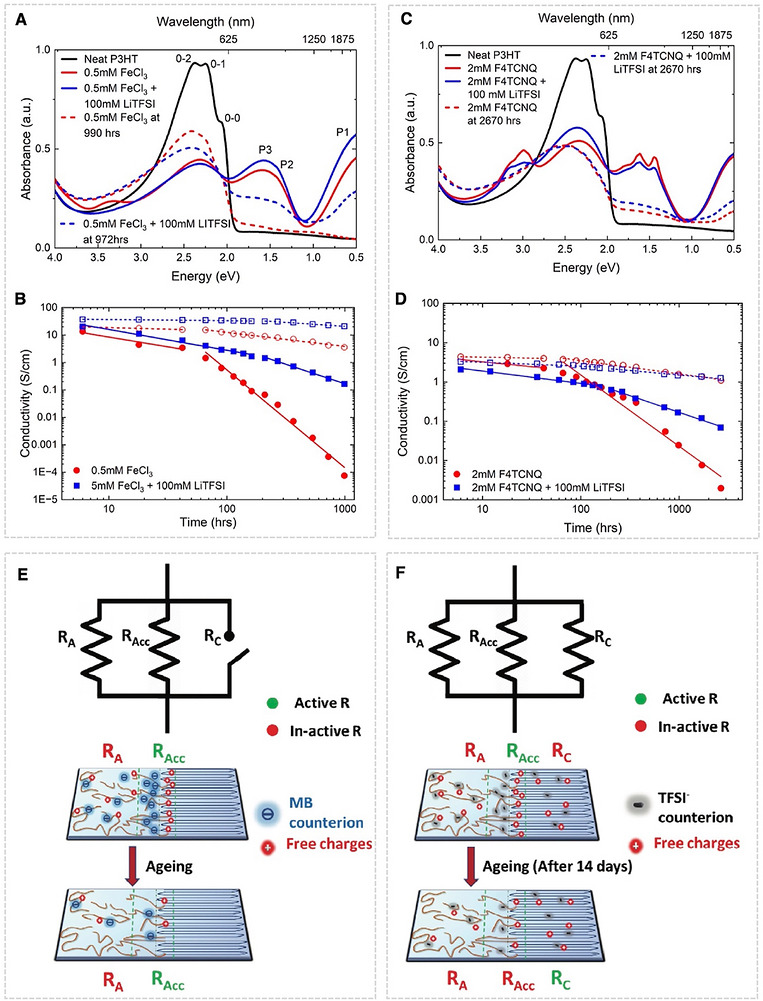
(A,C) UV−vis−NIR absorption spectra of P3HT neat (black), doped (solid), and dedoped in air (dashed); (B,D): Conductivity measurements of doped P3HT films over dedoping time [213]. “A–D) Adapted with permission [213]. Copyright 2024, American Chemical Society”. Illustration of relevant channels for charge transport in MB‐(E) and TFSI^−^(F)‐exchanged P3HT films by a parallel resistance model, and schematics illustrating the charge distribution in different channels [214]. “E,F) Adapted under the terms of the CC‐BY Creative Commons Attribution 4.0 International license (https://creativecommons.org/licenses/by/4.0) [214]. Copyright 2024, The Authors, published by Wiley‐VCH”.

It is generally believed that p‐type doped polymer films are more stable in air than n‐type doped ones. In fact, the stability of many p‐doped systems is also unsatisfactory. Moule et al. found that FeCl_3_, one of the most commonly used p‐type dopants, can induce detrimental radical side reactions that compromise the integrity of the polymer backbone, resulting in a decline of the electronic properties [213]. Note that upon exposure to air, the deterioration of the electronic properties occurs at a rate that is orders of magnitude greater. Although the concentration of [FeCl_4_]^−^ can be diminished through anion exchange doping, this does not guarantee an enhancement in doping lifetime, as the exchange electrolytes may function as co‐reactants in the degradation process (Figure 16A,B). In comparison, when F4TCNQ, another most commonly used p‐type dopant, is employed as the reactive dopant for doping, the initial conductivity is lower than that in the case of FeCl_3_‐doped; however, the lifetime of the doped polymer films is nearly three times that of the ones doped with FeCl_3_ (Figure 16C,D). As aforementioned, the magic blue was recognized as an efficient dopant that allows for modulation doping of P3HT and PBTTT, leading to extraordinarily high electrical performance [42]. Recently, a mechanistic investigation by the same groups of researchers indicated a compromise between high initial doped conductivity and long‐term film stability [214]. Although MB‐doped and TFSI‐exchanged P3HT films show comparable thermoelectric performances, their behavior during aging is markedly different. The larger MB counterions exclusively reside in the amorphous regions of the films. Over time, the dedoping effect leads to a fast and significant decline in the electronic properties (Figure 16E). In comparison, the smaller TFSI^−^ counterions are present in both crystalline and amorphous domains, and after aging, they remain in the crystalline regions for an extended period, accounting for the much better stability of the doped films (Figure 16F). This work underscores that the doping instability in the amorphous phase can result in a rapid decline in charge transport properties of the doped films, but the electrical performance after prolonged aging is determined quantitatively by the specific phase in which the charges are retained.

**FIGURE 17:**
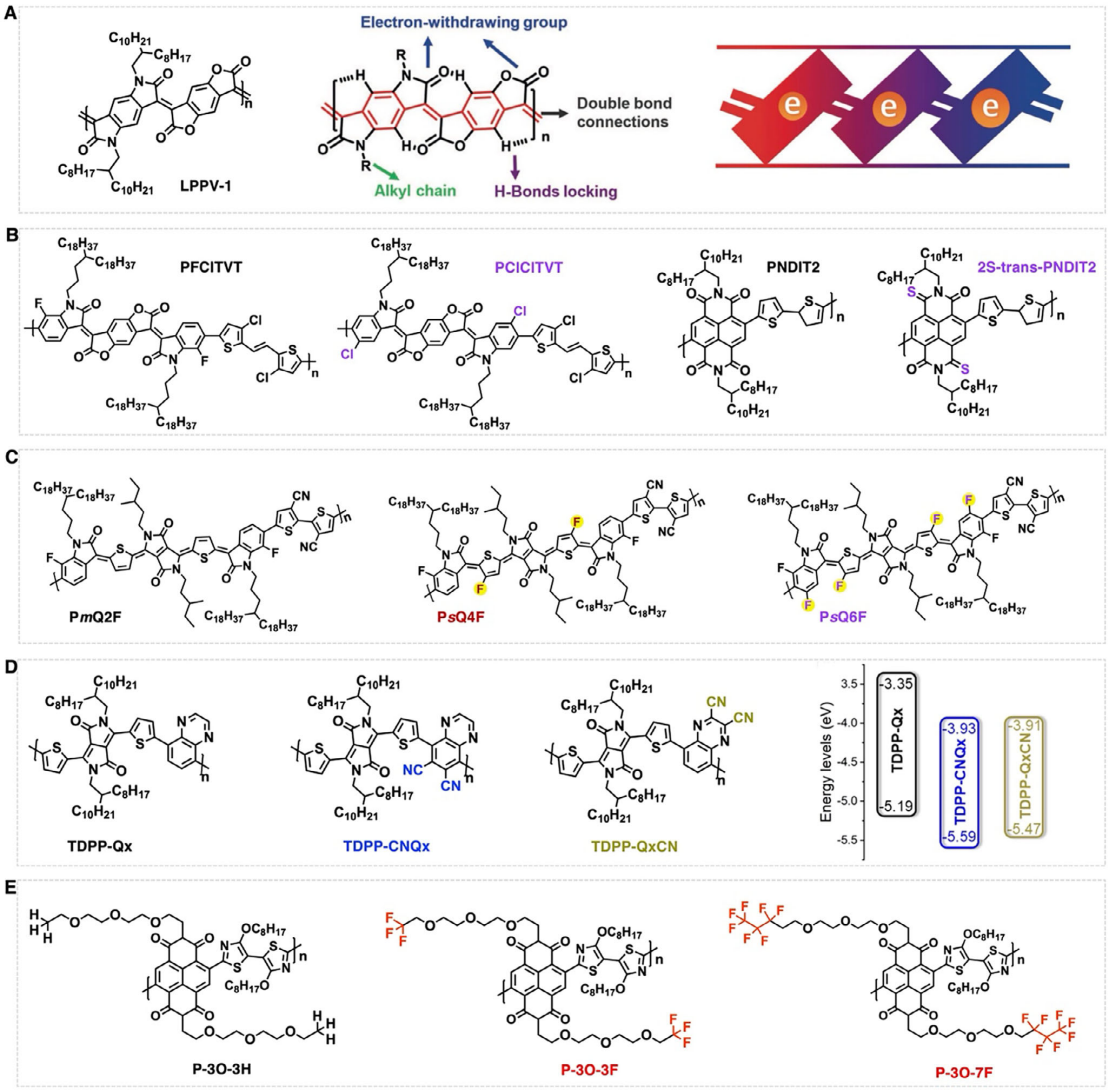
(A) Molecular structure of LPPV‐1, and illustration of its structural advantages for stable n‐type thermoelectrics. Adapted with permission [223]. Copyright 2019, Wiley‐VCH. (B) Chemical structures of PFClTVT, PClClTVT, PNDIT2, and 2S‐trans‐PNDIT2. (C) Chemical structures of PmQ2F, P*s*Q4F, and P*s*Q6F. (D) Chemical structures of TDPP‐Qx, TDPP‐CNQx, TDPP‐QxCN, and their corresponding energy levels. Right figure: Adapted with permission [227]. Copyright 2025, American Chemical Society. (E) Chemical structures of P‐3O‐3H, P‐3O‐3F, and P‐3O‐7.

Since negatively charged polarons are intrinsically reactive and sensitive to water, oxygen [215, 216, 217], and reactive oxygen species [218], the n‐doped materials typically suffer from low operational stability and poor air stability, resulting in decayed electrical performance of various devices in ambient conditions [50, 219]. Recently, much effort has been made to enhance the stability of n‐doped polymer films. One common strategy is developing novel conjugated polymers with deeper‐lying LUMO levels [220, 221, 222]. Recently, a few articles were published on the backbone engineering of conjugated polymers for achieving lower LUMO energy levels and thus superior ambient stability. For instance, highly rigid and planar polymer LPPV was synthesized (Figure 17A), and its unique structure not only facilitates charge transport but also significantly deepens the LUMO (as deep as −4.49 eV), resulting in exceptionally outstanding air stability, with a degradation in thermoelectric power factor of only 2% after continuous exposure to air for one week [223]. Recently, copolymerization of difluoro‐ and dichloro‐substituted BDOPV with weak donor (i.e., dichlorodithienylethene, CLTVT) resulted in two polymers, PFClTVT, PClClTVT, (Figure 17B), with the latter exhibiting significantly higher air stability thanks to its densely packed film structure with short π–π stacking distances [224]. Likewise, the thiolation of naphthalenediimide‐based copolymers resulted in much better ambient stability of the doped films [225] (Figure 17B). More recently, using an electron‐deficient building block, oxindole‐terminated quinoidal unit, three polymers were synthesized with different numbers of fluoride atoms, P*m*Q2F, P*s*Q4F, and P*s*Q6F [226] (Figure 17C). Notably, with increasing the numbers of fluoride from 2 to 4 and 6, the polymers become isometrically purer with deeper LUMO energy levels, leading to better electron mobility as well as greater air stability after n‐doping. Very recently, two novel, structurally straightforward cyanated moieties, CNQx, were designed and employed to copolymerize with thiophene‐flanked DPP, yielding TDPP‐QxCN and TDPP‐CNQx with markedly lower LUMO (>0.5 eV) than the reference polymer containing a non‐cyanated analogue [227] (Figure 17D). Remarkably, the refined microstructure of TDPP‐CNQx facilitates both efficient electron transport and dopant permeation, as well as exceptionally low thermal conductivity (0.1 W m^−1^ K^−1^), resulting in a notably high electron mobility of 1.3cm^2^ V^−1^ and unparalleled n‐doping efficiency with record thermoelectric metrics upon ambient processing. On the other hand, side chain engineering also contributes to improving the ambient stability of the n‐doped polymers. Recently, polar glycol ether side chains terminated with fluorinated alkyl chains were introduced in PNDI‐based polymers to boost both thermoelectric metrics and ambient stability [228] (Figure 17E). Other strategies involve developing air‐stable n‐type dopants [229], as well as stable doping approaches [230, 231]. These strategies contribute to improved air stability in n‐doped polymers to varying extent; however, complete resistance to atmospheric degradation remains elusive, as the thermoelectric properties of the reported n‐doped polymers all declined after extended periods of air exposure. For better air stability, deeper LUMO levels are required. Note that achieving complete stability against oxygen requires an even more challenging LUMO energy of ≤4.99 eV [35]. Thus, more electron‐deficient building blocks are required in the future.

**FIGURE 18:**
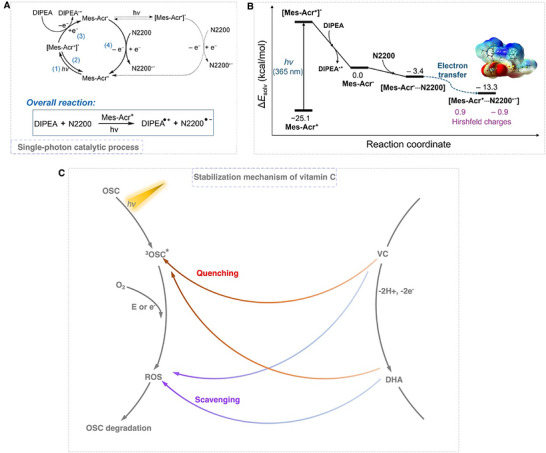
(A,B) Mechanism of n‐doping of N2200 under the photoredox condition, with the dotted line representing the unlikely “two‐photon–two‐electron” process. Adapted under the terms of the CC‐BY Creative Commons Attribution 4.0 International license (https://creativecommons.org/licenses/by/4.0) [232]. Copyright 2025, The Authors, published by American Association for the Advancement of Science. (C) Sketch of the stabilization mechanism of vitamin C. Concept based on ref [233].

Recently, a mild, air‐stable amine, which acts as a reducing agent, together with an air‐stable acridinium salt, which acts as a photoredox catalyst, were utilized to effectively dope common n‐type conjugated polymers, producing conductivity values comparable to the highest achieved through conventional doping methods [232]. Hereby, the photoredox n‐doping takes place through a one‐photon‐one‐electron transfer process, which is catalytic in essence (Figure 18A,B). Such a facile but efficient strategy expands the scope of n‐doping of organic semiconductors toward high‐performance and stable organic thermoelectrics. Recently, a versatile vitamin C (VC)‐based technique was reported to have a remarkable advantage both in stabilizing the n‐type OSCs (for instance, the benchmark n‐type molecule PTCDI‐C_8_) and enhancing the electrical performance of their corresponding devices, and it was successfully demonstrated in OFETs [233]. In principle, through a cascade process of sacrificial oxidation and non‐sacrificial triplet quenching, vitamin C is able to remove reactive oxygen species and prevent their formation, as shown in the illustration in Figure 18C. This process can shield the molecular structure from oxidation over time and simultaneously deactivate potential electron traps to stabilize electron transport. As shown in Figure 18D, under UV irradiation, the absorption peaks of PTCDI‐C_8_ gradually decrease because of photobleaching; remarkably, the degradation effect was significantly inhibited when VC was deposited on the film surface of PTCDI‐C_8_ (Figure 18E). Note that VC tends to crystallize and dewet on the substrate; polyurethane (PU) was used to blend with VC to facilitate solution processing for uniform film formation and to introduce flexibility to the film. When tested in OFET devices, VC‐PU significantly improved the stability and the performance of the devices. The performance of n‐type OFETs protected by vitamin C has been greatly improved, with electron mobility increased by up to 38 times, and operational stability and anti‐photooxidation ability greatly improved, and its excellent performance can be maintained in air for more than 255 days. This anti‐oxidation strategy shows excellent uniformity and batch repeatability in large‐area OFETs arrays. This study provides a new strategy for overcoming the stability issue of n‐type organic semiconductors. In another recent report, VC acts as a useful additive in organic thermoelectrics as it enables a simultaneous boost in electrical conductivity and the Seebeck coefficient of PEDOT:PSS, thanks to its innate polar nature and reducing ability [234]. To date, the effect of VC in improving n‐type organic thermoelectrics has yet to be investigated. Nevertheless, as a key material, vitamin C is environmentally friendly, low‐cost, and easy to use, holding huge potential for the future achievement of ambient‐stable polymer thermoelectrics and large‐scale industrialization.

### 6.2 Thermal Stability of Thermoelectric Polymers

**FIGURE 19:**
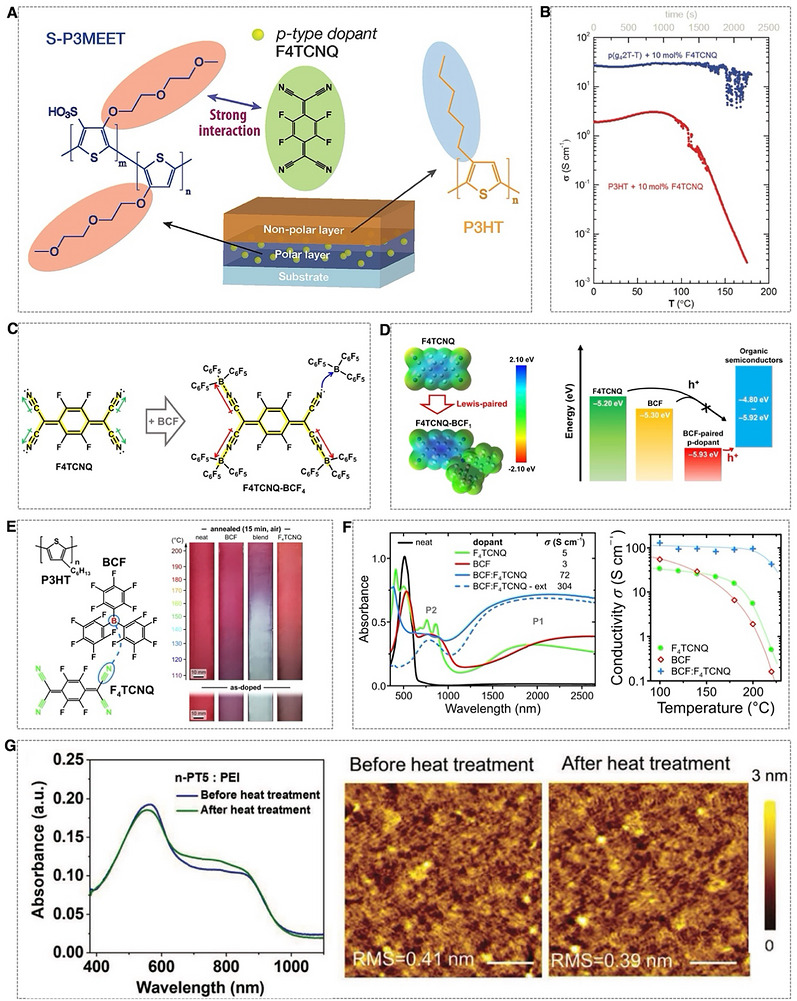
(A) Illustration showing that F4TCNQ binds with a substantially higher affinity to the polar S‐P3MEET than to non‐polar P3HT. Reproduced with permission [235]. Copyright 2016, Elsevier. (B) The thermal stability test shows that F4TCNQ‐doped polar p(g42T‐T) is more stable than F4TCNQ‐doped non‐polar P3HT. Reproduced under the terms of the CC‐BY Creative Commons Attribution 4.0 International license (https://creativecommons.org/licenses/by/4.0) [236]. Copyright 2017, The Authors, published by Wiley‐VCH. (C) Schematic representation of the structural changes in F4TCNQ upon Lewis pairing with BCF molecules. (D) ESP maps of neutral F4TCNQ and F4TCNQ‐BCF1; schematic representation showing the reduction potentials of several p‐dopants, HOMO levels of tested OSCs, and hole‐transfer processes from dopants to semiconductors. “C,D) Adapted with permission [237]. Copyright 2023, Wiley‐VCH”. (E) Transmitted‐light images of doped P3HT films following thermal annealing in air and (below) sections of the same films prior to annealing. (F) Absorption spectra and electrical conductivity values for P3HT films doped with the three dopant systems. “E,F) Adapted under the terms of the CC‐BY Creative Commons Attribution 4.0 International license (https://creativecommons.org/licenses/by/4.0) [76]. Copyright 2024, The Authors, published by American Chemical Society”. (G) UV–vis–NIR absorption spectra and AFM height images of PEI doped n‐PT5 films before and after heat treatment for 24 h. Adapted under the terms of the CC‐BY Creative Commons Attribution 4.0 International license (https://creativecommons.org/licenses/by/4.0) [239]. Copyright 2024, The Authors, published by Wiley‐VCH.

Low thermal stability has been a long‐lasting challenge for organic thermoelectrics. Take the benchmark p‐dopant F4TCNQ, for example: its tendency to significantly diffuse (after annealing at 80°C) out of the host matrix compromises the thermal stability of the doped P3HT polymers [235]. As observed (Figure 19A), F4TCNQ binds with a substantially higher affinity to the polar S‐P3MEET than to non‐polar P3HT [235]. After heating at 210°C, F4TCNQ was largely retained in the polar layer. By contrast, F4TCNQ was more unstable in the non‐polar layer, even diffusing out at ambient temperatures. Remarkably, when the polar p(g42T‐T) was doped with F4TCNQ, the films were highly thermally stable at temperatures up to 150°C [236] (Figure 19B). Recently, Lewis‐paired CN groups were developed as a novel class of building blocks for efficient and stable doping [76, 237]. As shown in Figure 19C, one F4TCNQ molecule is able to coordinate with up to four BCF molecules, leading to extremely electron‐withdrawing properties (Figure 19D), as confirmed by spectroscopic analysis and DFT calculations [237]. Such large‐sized Lewis‐paired dopants allow for substantially suppressing molecular thermal diffusion, and a simultaneous outstanding doping efficiency, resulting in highly stable and efficient doped films [76] (Figure 19E,F). Moreover, polymer host/polymer dopant is another effective strategy for more stable thermoelectric devices [238, 239]. For instance, the PEI‐doped n‐PT5 is thermally stable, and its morphology is not significantly altered before and after heat treatment [239] (Figure 19G).

## 7 Unconventional Applications of Thermoelectric Polymers

The unique properties of thermoelectric polymers, such as printability, flexibility, and even stretchability, endow them with advantages over their inorganic counterparts [240, 241]. However, as discussed in the preceding sections, the transition from materials to functional devices is fundamentally driven by the advanced doping processes and the control of charge transport in complicated ambient conditions, where the performance of the devices is to a great extent dictated by the design of high‐performance polymers. These mechanistic advances allow thermoelectric polymers to be applied to several unconventional fields, such as printed thermoelectrics, thermoelectric textiles, thermoelectric elastomers, and various sensors. Particularly, the rapidly growing need for wearable devices and IoT makes thermoelectric polymers more and more desirable.

### 7.1 Printed and Flexible Organic Thermoelectrics

**FIGURE 20:**
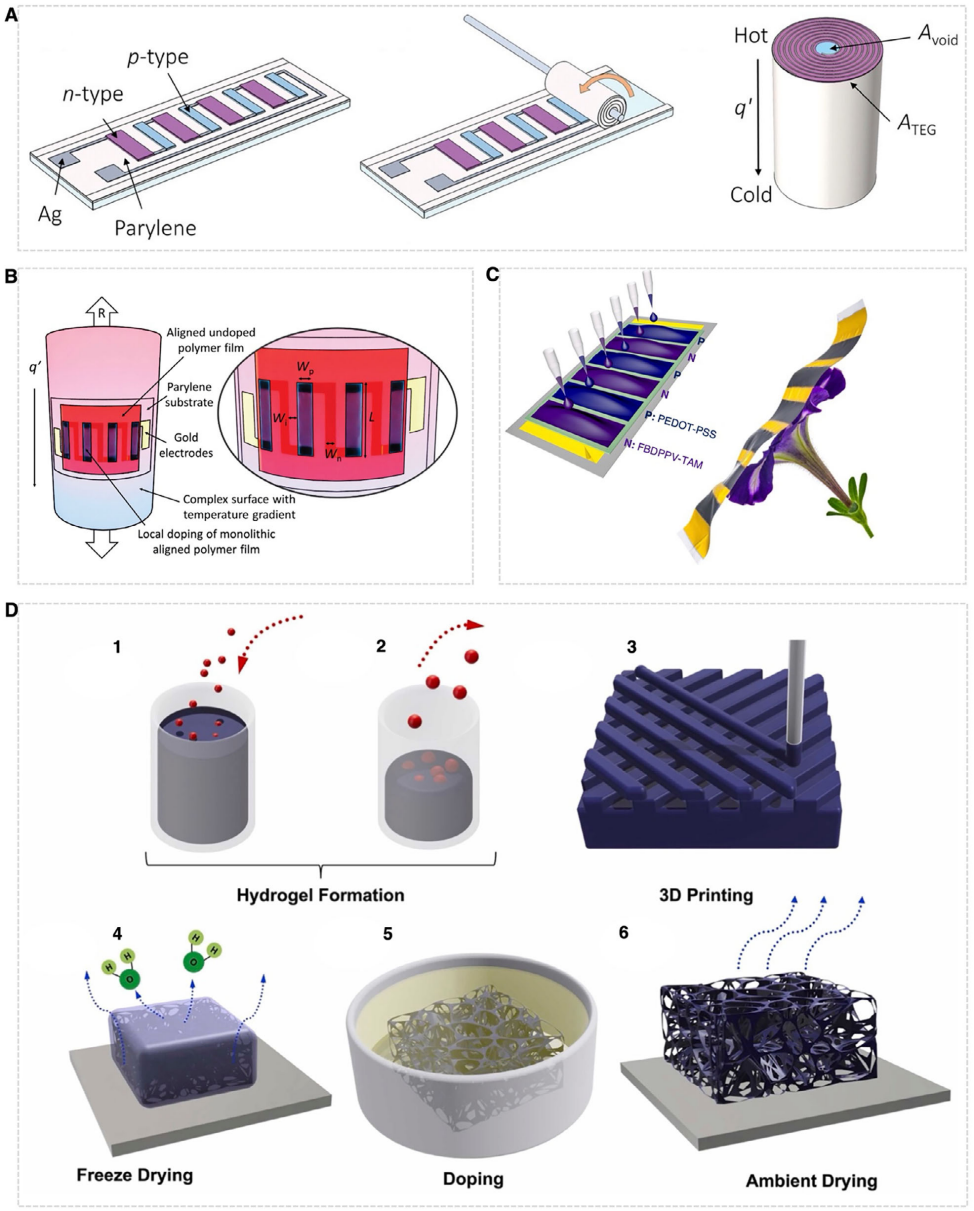
(A) Depiction of the high‐density µTEG architecture after planar fabrication, and rolling of the delaminated µTEG to achieve its high‐density orientation. Adapted under the terms of the CC‐BY Creative Commons Attribution 4.0 International license (https://creativecommons.org/licenses/by/4.0) [242]. Copyright 2024, The Authors, published by Wiley‐VCH. (B) Depiction of the conformable aligned TEG on a hypothetical surface with an existing temperature gradient. Adapted under the terms of the CC‐BY Creative Commons Attribution 4.0 International license (https://creativecommons.org/licenses/by/4.0) [245]. Copyright 2025, The Authors, published by Wiley‐VCH. (C) Flexible all‐polymer solution‐processed thermoelectric generator based on PEDOT‐PSS as p‐legs and TAM‐doped FBDPPV as n‐legs. Adapted under the terms of the CC‐BY Creative Commons Attribution 4.0 International license (https://creativecommons.org/licenses/by/4.0) [246]. Copyright 2020, The Authors, published by Springer Nature. (D) Six‐step sample fabrication process of 3D printed PEDOT: PSS aerogel. Adapted with permission [249]. Copyright 2023, Elsevier.

The solution‐solubility of thermoelectric polymers endows them with the suitability for scalable printing and patterning approaches. For instance, a rolled µ‐TEG was fabricated by integrating large‐area inkjet printing techniques with an ultrathin parylene substrate [242]. Such a process involves the deposition of p‐ and n‐legs, as well as the electrodes on the flexible substrate, followed by rolling the delaminated µTEG to achieve a high‐density orientation (Figure 20A). Note that for practical applications, it is very important to achieve high power output density [243, 244]. The as‐fabricated µTEG demonstrated a power output of 0.15µW cm^−2^ at a temperature gradient of 50 K.

Emerging doping physics has enabled the patterning of devices with much greater spatial precision. Specifically, aligned polymer films were transferred onto ultrathin parylene substrates and patterned using a local inkjet doping approach, resulting in monolithic TEGs with an exceptional power factor of approx. 1 nW cm^−2^ K^−2^ [245] (Figure 20B). Finally, a thermoelectrically‐powered volume‐indicating label was demonstrated, showing potential applications in the healthcare and food industries. Notably, all‐polymer TEGs were successfully fabricated by drop‐casting PEDOT:PSS as p‐legs and TAM‐doped FBDPPV as n‐legs [246] (Figure 20C). The as‐fabricated TEGs are both flexible and light, which yield 0.18 nW at a hot‐side temperature of 30°C with a temperature gradient of only 2.3 K. During the past few years, 3D printing has developed very rapidly, and it has been successfully applied in the fabrication of organic electronics [247, 248]. Recently, 3D printing with a tertiary doping process was employed to fabricate polymer aerogel‐based TEGs, enabling the printing of PEDOT:PSS aerogel with well‐controlled geometry [249] (Figure 20D). The printed aerogel achieved a fourfold increase in thermoelectric output compared to the pristine aerogel. This work represents a new avenue for enhancing the thermoelectric performance of conducting polymers by uncovering their intrinsic properties, thereby reducing the reliance on external additives. Note that optimizing the dopant‐polymer interaction allows the intrinsic transport properties of the polymers to dictate the device efficiency. More studies on printed thermoelectric polymers can be found in a few recent review articles [20, 202].

### 7.2 Thermoelectric Textiles for Wearable Devices

**FIGURE 21:**
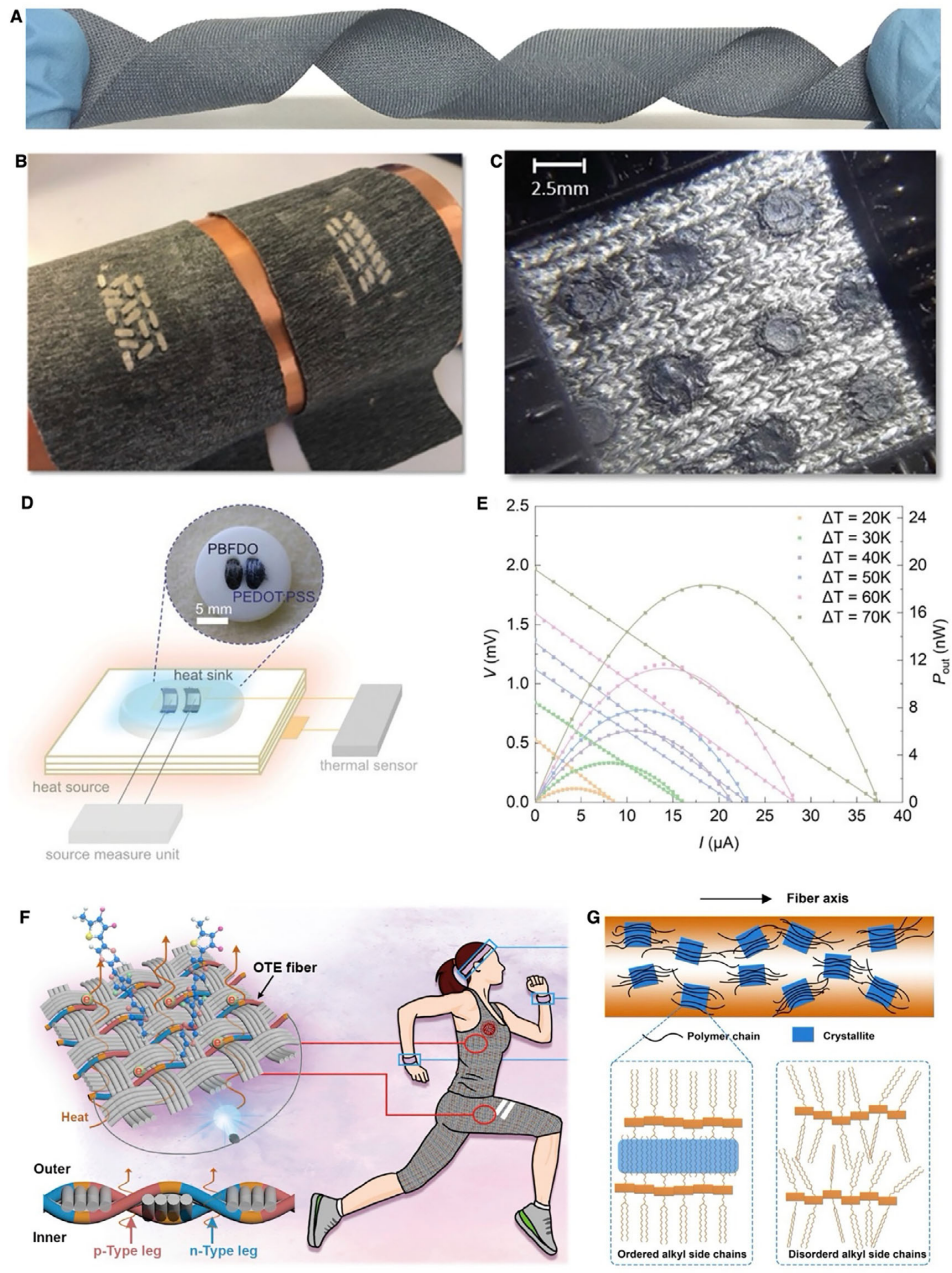
(A) digital photo of polyester fabric after coating treatment with PEDOT: PSS. Adapted under the terms of the CC‐BY Creative Commons Attribution 4.0 International license (https://creativecommons.org/licenses/by/4.0) [251]. Copyright 2015, The Authors, published by Springer Nature. (B) Wearable TEG integrated into knitted fabric consisting of 32 p‐ and n‐type legs arranged in a hexagonal close‐packed layout and connected according to the Hilbert curve. (C) Uneven topographies were observed on the textile TEG after the printing and drying process. (D) Schematic representation of the thermocouple fabricated by hand‐stitching a button onto a wool fabric with the n‐type leg composed of PBFDO‐coated yarn and the p‐type leg of PEDOT: PSS‐coated yarn. (E) The performance of the thermoelectric button by placing the device between a hot plate and a heat sink with different temperature gradients. (F) Schematic diagram of potential wearable applications of the semiconducting polymer fibers. (G) Schematic diagram of the mechanism of the mechanical strength and stretchability enhancement of the semiconducting fibers. “B,C) Adapted with permission [252]. Copyright 2019, Wiley‐VCH. D,E) Adapted under the terms of the CC‐BY Creative Commons Attribution 4.0 International license (https://creativecommons.org/licenses/by/4.0) [254]. Copyright 2024, The Authors, published by Wiley‐VCH. F,G) Adapted under the terms of the CC‐BY Creative Commons Attribution 4.0 International license (https://creativecommons.org/licenses/by/4.0) [77]. Copyright 2024, American Association for the Advancement of Science”.

To harvest body heat for powering wearable devices, integrating polymeric TEGs into textiles is an appealing method [250]. Thus, textile‐compatible fabrication is essential. Further, the transition from rigid substrates to soft fabrics requires the maintenance of high charge mobility and thus high conductivity within the irregularly shaped fiber networks. The initial attempts at fabricating thermoelectric fabrics started a decade ago with PEDOT:PSS [251]. As shown in Figure 21A, the PEDOT:PSS‐coated polyester fabric is flexible, and its breathability is not affected by the coating. A 5‐strip flexible TEG device is able to produce 4.3 mV and 12.3 nW at a temperature gradient of 75.2 K. Notably, the first proof‐of‐concept for a textile TEG using both p‐ and n‐type conducting polymers was achieved using stencil and transfer printing on a commercial sports fabric, which is capable of capturing “through‐plane” body heat [252] (Figure 21B). The inks were formulated from PEDOT:PSS as p‐type and Poly[Na(NiETT)] as n‐type polymers. Remarkably, A 32‐leg test device produced an open‐circuit voltage of 3 mV at a temperature difference of 3 K, and an 864‐leg device was able to generate 47 mV. Nevertheless, morphological investigation indicates that after printing and drying, uneven topographies were observed (Figure 21C), due primarily to the fact that p‐type ink tended to form a concave surface, whereas the n‐type ink exhibited significant shrinkage and crack formation. Such topographies are responsible for high contact resistance. Solving this problem requires more than just better protocols, but it necessitates the development of efficient n‐type materials not only with better ability for superior ink formulation, but also with intrinsic structural integrity to withstand drying stresses. With the emergence of solution‐processable, highly conductive PBFDO, the field has addressed the aforementioned problem through molecular design that favors dense packing and stability, and correspondingly, better n‐type inks have also been prepared. Recently, n‐type polymer fiber have been produced through the wet‐spinning of PBFDO, leading to a high electrical conductivity over 1000 S cm^−1^ [253]. Such an outstanding performance is a direct application of the high‐conductivity n‐type polymers discussed in Section 2. Notably, even in a high‐humidity environment, fiber conductivity stabilizes after 2 weeks and retains 81% of its initial conductivity. For proof‐of‐concept textile TEGs, bundles of PBFDO (n‐type) and PEDOT:PSS (p‐type) fibers were stitched together to form a two‐couple thermoelectric textile [253]. Later, PBFDO was further investigated in yarn coating and demonstrated remarkable durability and ambient stability [254] (Figure 21D). Out‐of‐plane thermoelectric button and 16‐legged thermopile were fabricated using silk yarns coated with PBFDO and PEDOT:PSS, leading to notable performance and demonstrating PBFDO as a promising candidate for the coating of silk yarn [254] (Figure 21E).

Very recently, a flow‐enhanced crystallization (FLEX) method has been developed, which enables the continuous fabrication of conjugated polymer fibers with exceptionally high strength and toughness (Figure 21F). This technique represents a breakthrough in bridging fluid mechanics and solid‐state transport physics. Remarkably, the as‐fabricated fibers exhibit a tensile strength and toughness that are one order of magnitude greater than traditional semiconducting polymers, metrics that even outperform many synthetic fibers. This scalable process yields fibers with unique strain‐enhanced electronic properties, owing to the stronger resistance to sliding between lamellae (Figure 21G). This work uncovered the critical impact of fluid mechanics on the crystallization and mechanical properties of conjugated polymers, specifically the suppression of disorder during fiber formation, thus holding huge potential for future advanced thermoelectric textiles.

### 7.3 Thermoelectric Elastomers

**FIGURE 22:**
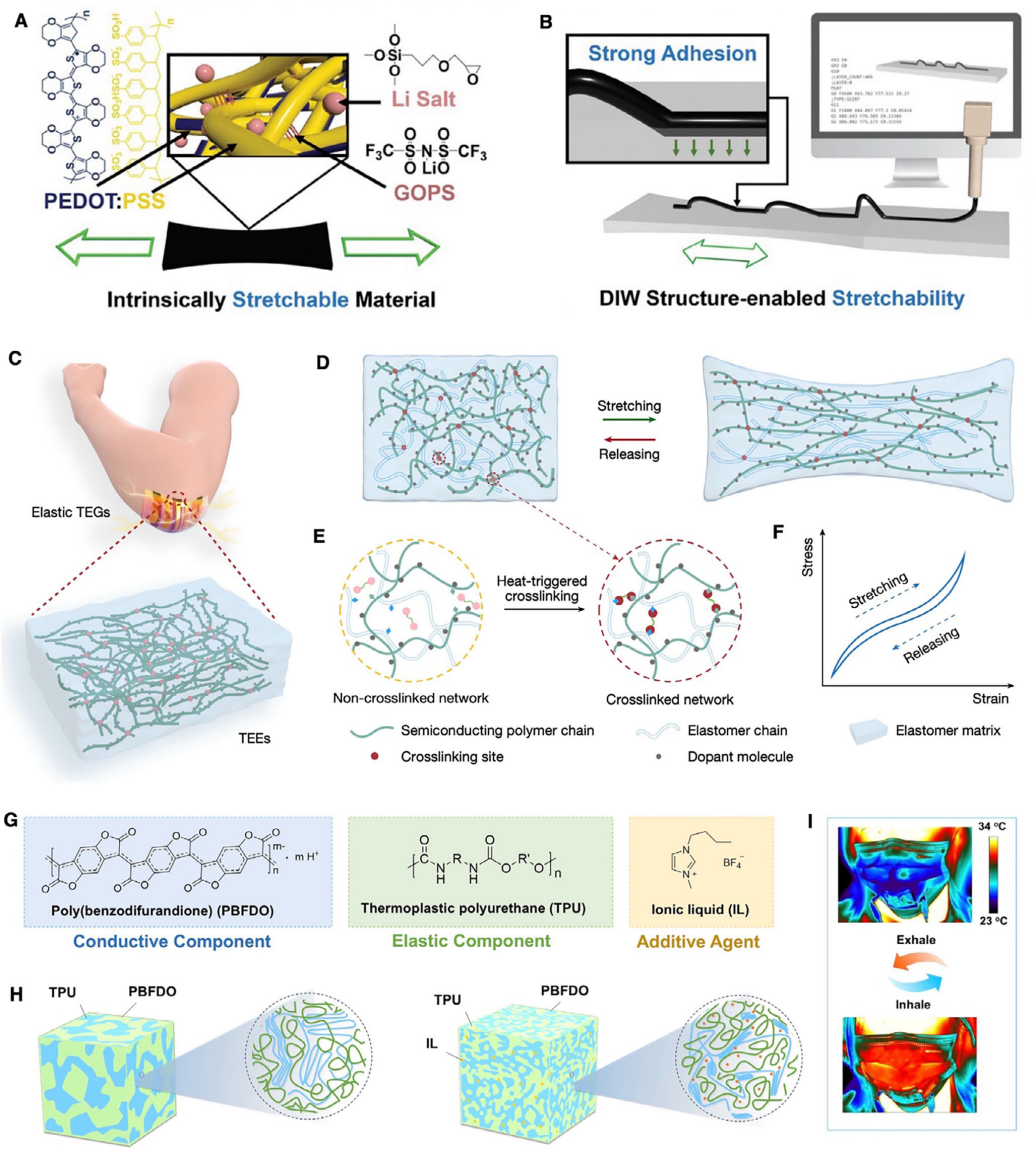
(A) Intrinsically stretchable PEDOT: PSS is achieved by incorporating GOPS (crosslinker) and Li salt (plasticizer). (B) Digital fabrication of PEDOT: PSS out‐of‐plane structures by DIW provide additional stretchability to the design. (C) Schematic of an elastic TEG made by TEEs attached conformably to a bent human elbow. (D) Schematic of the size change of a TEE during the stretching–releasing process and (E) the thermally activatable crosslinking mechanism. (F) Typical stress–strain curve. (G) Chemical structures of PBFDO, TPU, and IL. (H) Schematic diagrams representing the morphologies of the PBFDO/TPU film and the PBTI. (I) Infrared images during respiration. “A,B) Adapted under the terms of the CC‐BY Creative Commons Attribution 4.0 International license (https://creativecommons.org/licenses/by/4.0) [263]. Copyright 2025, The Authors, published by Wiley‐VCH. C‐F) Adapted with permission [74]. Copyright 2025, Springer Nature. G‐I) Adapted with permission [79]. Copyright 2025, Wiley‐VCH”.

To date, extensive efforts have been devoted to developing flexible thermoelectric polymers [255, 256]. To achieve wearable organic thermoelectric devices, however, materials with flexible features alone are not sufficient. Intrinsically elastic materials that can maintain efficient electronic pathways under significant strain are highly desired [257, 258, 259]. In particular, a deep understanding of the impact of doping on the mechanical properties of the polymer host is essential to address the stretchability of thermoelectric polymers [260]. Previously, a few studies focused on the development of stretchable p‐type thermoelectric elastomers based on PEDOT‐derived polymers [261, 262]. Notably, omnidirectional direct ink writing (DIW) was recently employed to successfully process intrinsically stretchable PEDOT:PSS aerogels, as a pathway to creating 3D soft thermoelectrics [263] (Figure 22A,B). This process involves formulating PEDOT:PSS hydrogels that are 3D printed and then freeze‐dried directly onto stretchable substrates. Such an in situ fabrication method yields integrated aerogel structures maintaining high shape fidelity with little shrinkage, which was demonstrated by creating 3D stretchable interconnects, planar TEGs, as well as vertical TE pillars. Nonetheless, the development of n‐type thermoelectric elastomers has been a major bottleneck due to both the lack of efficient n‐type materials and the undesired disruption of n‐type dopants on the polymer microstructure, and thus, it has been rarely reported. Recently, Lei et al. overcame the challenge of n‐type elasticity via fabricating intrinsically elastic TEGs by developing new materials combining bulk nanophase separation, thermally activated crosslinking, as well as targeted doping [74] (Figure 22C–F). Specifically, carefully chosen dopant and elastomer facilitate the formation of an ideal nanostructure where highly n‐doped semiconducting polymer nanofibrils are wrapped and isolated by the elastomeric matrix (Figure 22D,E). Such a scenario allows for concurrently enhancing electrical characteristics via the percolating nanofibril network while reducing thermal characteristics through the insulating matrix, yielding an elastomer with notably high ZT values even when stretched up to 150% [74]. Almost simultaneously, another study was published that focuses on fabricating n‐type thermoelectric elastomers by synergistically blending the benchmark n‐type PBFDO as the conductive component, stretchable TPU as the elastic component, and an ionic liquid as a mediator [79] (Figure 22G,H). The designed composites exhibited a remarkable n‐type conductivity over 200 S cm^−1^, fracture elongation above 200%, and long‐standing operation. When combined with p‐type PEDOT:PSS, a stretchable TEG was fabricated, and it was successfully used in a fire safety alarm system, as well as serving as a real‐time human physiological monitor (Figure 22I). These recent studies demonstrate that the historical hurdle of stability and performance in elastomers is being solved by sophisticated control over the spatial distribution of the dopants and morphology of the materials.

### 7.4 Thermoelectric Polymers for Sensing Applications

**FIGURE 23:**
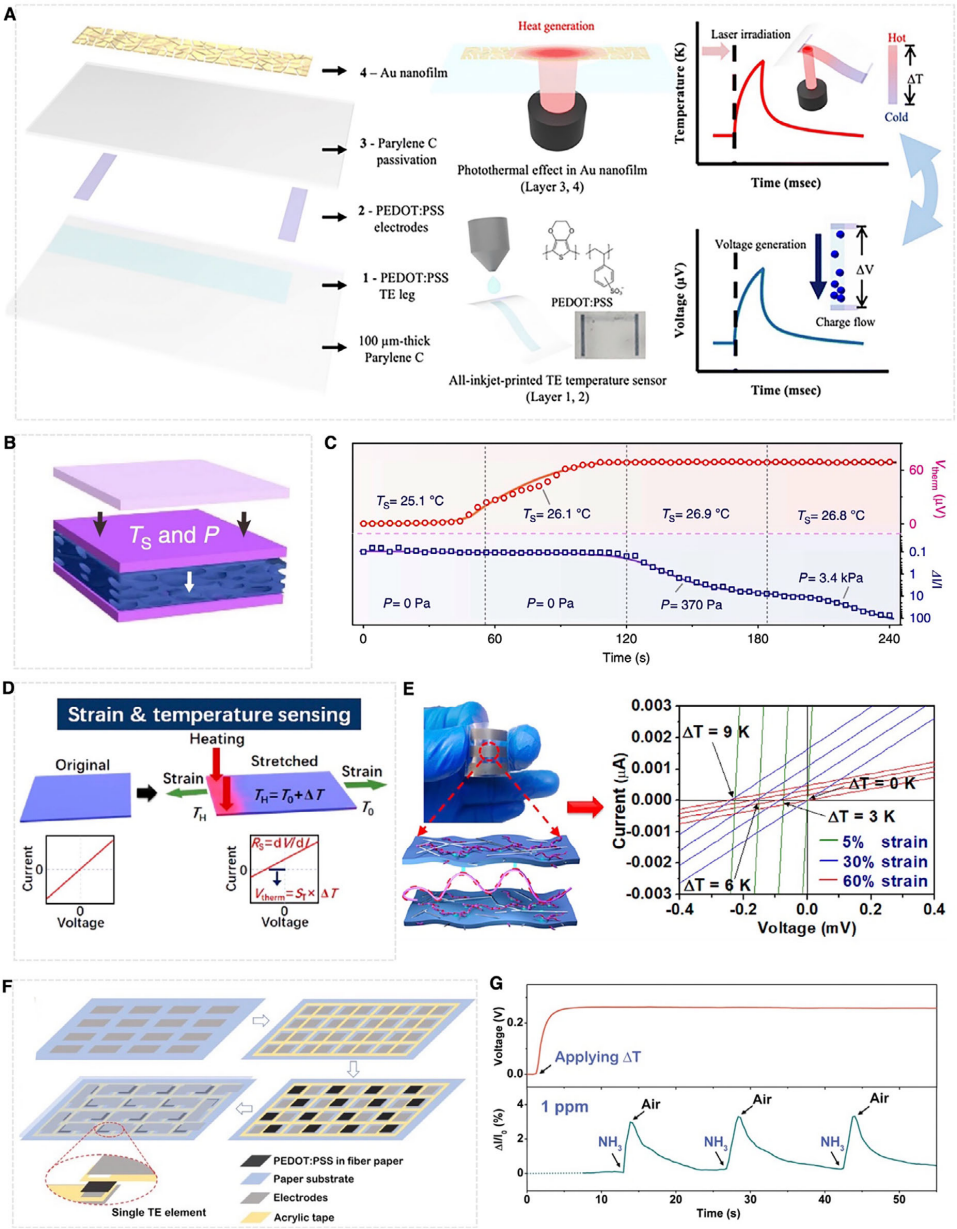
(A) Conceptual illustration of high temporal resolution of the all‐inkjet‐printed TE transparent temperature sensor and its application to photothermal effect sensing. (B) Loading of a coupled temperature and pressure stimulus on the sensor. (C) Plots of the real‐time output voltage and current responses of an MFSOTE device approached by a Peltier element. (D) Loading of a coupled strain and temperature stimuli on the sensor. (E) Illustration of the film structure of the sensor (left) and measured I−V curves of the nanocomposite sensing device taken under different ΔT and various applied strains. (F) Schematic illustration of the fabrication process of an OTE generator with PEDOT: PSS legs for sensing. (G) Output voltage of an OTE array driven by a temperature difference created by a heating plate and a cooling flow, and the time monitoring of the current change in response to 1 ppm ammonia of the sensing OFET powered by the OTE array. “A) Adapted with permission [264]. Copyright 2023, Royal Society of Chemistry. B,C) Adapted under the terms of the CC‐BY Creative Commons Attribution 4.0 International license (https://creativecommons.org/licenses/by/4.0) [265]. Copyright 2015, The Authors, published by Springer Nature. D,E) Adapted with permission [266]. Copyright 2020, American Chemical Society. F,G) Adapted with permission [267]. Copyright 2019, Wiley‐VCH”.

Sensors serve as a foundational technology in modern society, as they act as the critical link between the physical world and digital data systems, enabling the conversion of a wide range of stimuli into measurable electrical signals. Owing to the Seebeck effect, thermoelectric polymers have been widely applied in the sensing of temperatures, as they can translate thermal gradients directly into electrical signals. Compared to conventional metallic thermocouples, the polymer‐based materials can be biocompatible and optically transparent, making them ideal materials for human‐body temperature detection and even for tissue integration. Particularly, in modern medicine and healthcare, it is a fundamental requirement that the rapid and localized temperature shifts induced by the photothermal effect must be accurately measured. In the past, however, sensors have failed to capture these thermal dynamics, due primarily to a poor optical transparency and a sluggish response time. Moreover, metallic probes tend to block the light path and absorb incident radiation, resulting in artifacts in the collected signals. Better transparency and higher response speeds are therefore desired. Recently, high‐speed temperature sensors were fabricated by low‐cost and scalable inkjet‐printing of biocompatible thermoelectric polymers [264] (Figure 23A). This high‐speed response allows for the first time the capture of transient thermal gradients that would be missed by conventional equipment. Note that these sensors are optically transparent, making them essentially insensitive to direct laser irradiation, which guarantees high thermoelectric sensitivity by avoiding artifacts caused by the light‐sensor interactions. Remarkably, these sensors were able to monitor photothermal phenomena with a resolution of microseconds, making them highly promising for future photothermal research and biomedical diagnostics.

With the increasing number of parameters to be monitored in modern society, sensor architectures have to be increasingly complex and are thus difficult to manufacture using traditional methods. To mitigate the manufacturing complexities, novel materials capable of reacting to multiple stimuli within a single platform are highly desired. Thermoelectric polymers are uniquely positioned to address this need because they can serve as multifunctional transducers, thereby reducing the reliance on multi‐component assemblies. For instance, the first flexible dual‐functional sensor capable of simultaneous temperature and pressure detection was developed through depositing PEDOT:PSS onto polyurethane (PU) foam via dip coating [265] (Figure 23B,C). The temperature fluctuations are detected by monitoring the voltage shift in the IV curve, a direct application of the Seebeck effect, while the pressure stimuli are quantified through resistance variation in the *I–V* curve, induced by the mechanical deformation of the PU structure. Moreover, a stretchable dual‐functional sensor capable of simultaneous temperature and strain detection was developed [266] (Figure 23D). Notably, it can precisely detect and distinguish strain from temperature stimuli without crosstalk (Figure 23E). In addition, a flexible self‐powered sensing element was developed by integrating organic‐transistor‐based chemical sensors with a flexible power source, an organic thermoelectric generator [267] (Figure 23F). Specifically, the OTE array was fabricated on a versatile paper substrate, achieving a peak open‐circuit voltage of 0.52 V and a maximum power output of 0.32 µW. Significantly, this output is sufficient to drive OFET gas sensors characterized by ultralow operating voltages. The resulting integrated system demonstrates high sensitivity in ammonia detection without the requirement of an external power supply (Figure 23G). In these applications, the thermoelectric polymers act as both the active sensing material and the transducer, converting external stimuli directly into measurable voltage signals. As a result, the growing synergy between polymer science and device engineering is positioning polymer thermoelectrics as a cornerstone technology for the IoT and the next generation of autonomous, wearable electronics.

## 8 From Laboratory to Real‐World: Translational Challenges

While polymer thermoelectrics have reached a new, transformative era, the aforementioned breakthroughs remain largely confined to laboratory‐scale demonstrations. The translation of these high‐performance thin films into real‐world applications remains a significant challenge, depending upon future advancements in cost‐effective scalability, process compatibility, long‐term operational stability, and seamless system integration.

While the constituent elements of conjugated polymers are a lot more abundant than those of inorganic alloys, the commercial viability of high‐performance polymers is often constrained by the ultra‐high costs of specialized monomers and sophisticated dopants. Specifically, many state‐of‐the‐art polymers described in Section 2 involve complicated synthetic routes, e.g., Stille or Suzuki coupling, which necessitate expensive palladium catalysts and high‐purity toxic monomers. Moreover, most sophisticated architectures require multistep, low‐yield syntheses, further increasing the cost‐per‐watt [268]. Beyond direct costs, conventional synthesis often suffers from significant batch‐to‐batch variability, since both Mw [269, 270] and the polydispersity index (PDI) [271] play non‐negligible roles in determining the final electronic properties. Consequently, the cumulative expenses associated with high‐purity precursors, costly catalysis, and labor‐intensive processing can easily drive materials costs into the thousands of €/gram range. As a result, achieving ultimate industrial scalability remains both a technical and a financial bottleneck.

**FIGURE 24:**
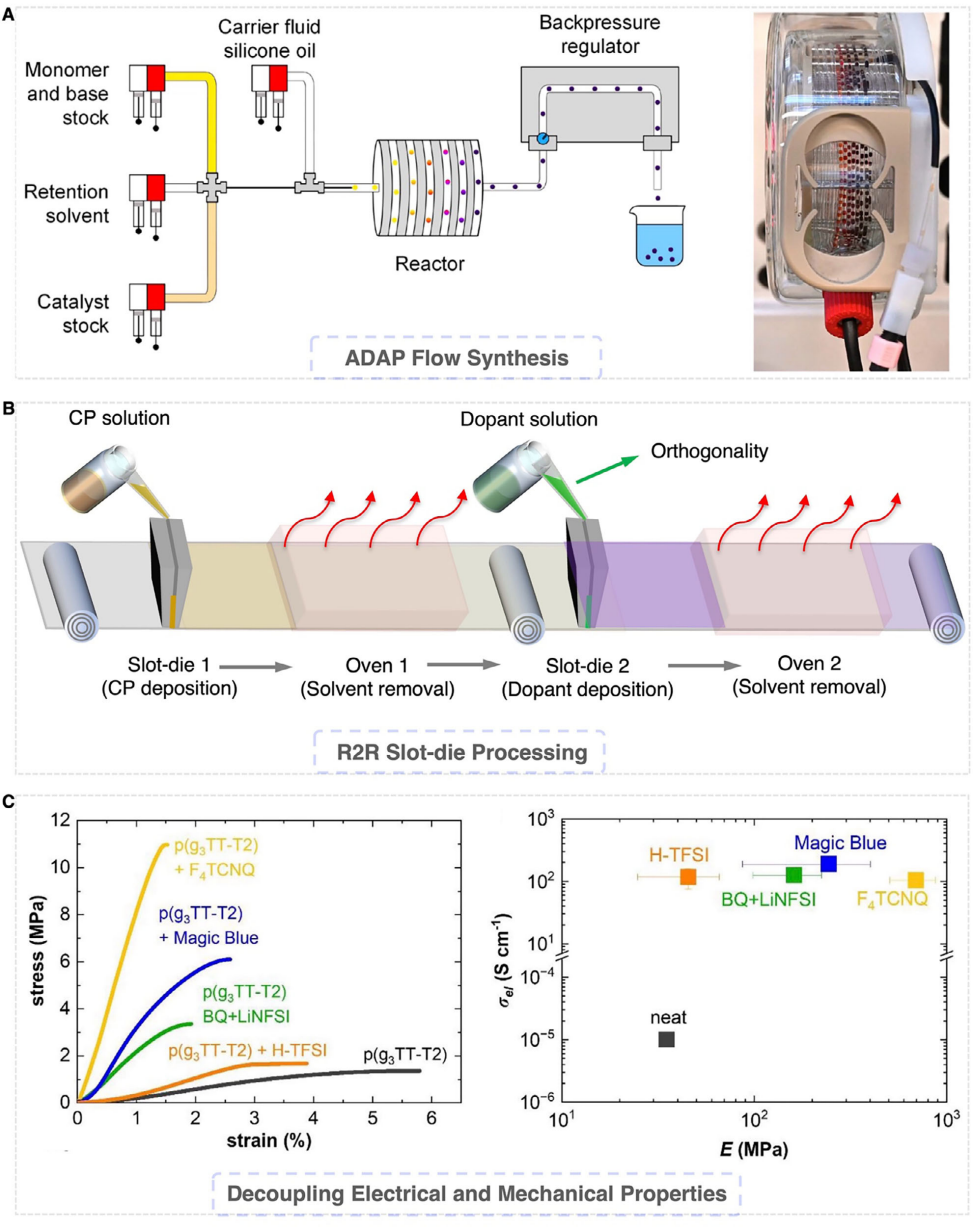
(A) Translation of polymerization from batch to flow synthesis: (left) schematic of flow synthesis setup; (right) image of NMP/H_2_O reaction droplets inside silicone oil in the reactor. Adapted under the terms of the CC‐BY Creative Commons Attribution 4.0 International license (https://creativecommons.org/licenses/by/4.0). Copyright 2025, The authors, published by American Association for the Advancement of Science. (B) Schematic illustration of R2R Slot‐die Processing for the fabrication of polymer thermoelectrics. (C) Decoupling the electrical and mechanical properties of conjugated polymers. Left: representative stress‐strain tensile deformation curves of free‐standing films of neat and doped p(g3TT‐T2) recorded at room temperature; Right: electrical conductivity σ*_el_* vs. Young's modulus E of neat and doped p(g3TT‐T2). Adapted under the terms of the CC‐BY Creative Commons Attribution 4.0 International license (https://creativecommons.org/licenses/by/4.0) [275]. Copyright 2026, The Authors, published by Wiley‐VCH.

To overcome these synthetic and financial barriers, encouragingly, recent research has been shifting toward more sustainable synthetic paradigms. For instance, ambient direct arylation polymerization (ADAP) has been developed [272], which avoids the requirement for costly organometallic monomers and toxic stannylated precursors, significantly reducing the synthetic complexity [273]. As demonstrated by Müller et al., the ADAP remarkably enables the open‐flask synthesis of a multitude of conjugated polymers at room temperature. This represents a successful translation of polymerization from batch to flow synthesis (Figure 24A), allowing scalability to over 100 grams, thereby significantly lowering the barrier to industrial‐scale production [272]. In addition, the transition to aqueous‐based synthesis and catalyst‐free oxidative polymerization not only mitigates the reliance on precious metals, like palladium, but also simplifies purification processes, paving a viable path toward affordable, large‐area polymer thermoelectric modules. While ADAP has been successfully demonstrated on structurally simpler systems, such as several polythiophenes and a DPP‐based derivative, its application to the increasingly complex architectures (e.g., multi‐component D‐A copolymers) of state‐of‐the‐art thermoelectric polymers remains an open question requiring further investigation.

On the other hand, optimizing processing methodologies represents a critical pathway toward reducing the cost‐per‐watt of polymer thermoelectrics. At the laboratory scale, conventional spin‐coating techniques are obviously inefficient, often resulting in the loss of the majority of the precursor solution during deposition. In contrast, transitioning to high‐throughput manufacturing, such as Roll‐to‐Roll (R2R) processing (Figure 24B), significantly enhances material utilization, thereby achieving utilization rates approaching 100%. Such a shift from centrifugal casting to continuous coating not only optimizes material consumption but also enables the exponential cost reductions, which is necessary for the commercial viability of polymer thermoelectrics. Since a comprehensive analysis of the latest advancements in R2R printing for organic thermoelectrics falls outside the primary scope of this work, the reader is encouraged to consult the most recent, in‐depth review by Molina‐Lopez, Caironi, and coworkers, dedicated specifically to the fabrication of printed organic thermoelectric generators [274].

Another bottleneck that prevents the transition of polymer thermoelectrics from laboratory demonstrations to commercially viable devices is process compatibility. The first challenge is the orthogonality constraint [276, 277]. During the fabrication of alternating p‐type and n‐type architectures, the deposition of the subsequent film often leads to the partial dissolution or swelling of the first/underlying film. Particularly, it is exceptionally difficult to achieve solvent orthogonality when both types of polymers share similar backbone structures. Such a limitation is not confined merely to the active layers, but also applies to the processing of dopants in sequential doping strategies, since the dopant solution must be carefully engineered to penetrate the polymer matrix, such that the doping facilitates charge transfer without causing disruption to the film morphologies. The second challenge stems from the discrepancy between laboratory‐level processing (typically centrifugal spin‐coating) and industrial R2R processing, as the former allows for fast solvent evaporation, whereas the latter involves distinct shear forces and slower drying kinetics, which may lead to different molecular packing orientations, most probably resulting in inferior thermoelectric performance. In essence, a polymer compatible with lab‐scale processing is not necessarily compatible with large‐scale industrial manufacturing. Consequently, solving these issues necessitates balancing many parameters, including solubility, interfacial interactions, and stable doping during the processing. One emerging strategy is the development of polymers with cleavable side chains, as discussed in Section 4.3, which allows for completely changing the solubility of polymers after side‐chain removal, such that the film becomes insoluble for subsequent layers. Finally, shifting from halogenated solvents toward greener, aqueous or alcohol‐based formulations will be essential for future process compatibility and environmental sustainability.

Beyond the aforementioned economic and processing challenges, long‐term operational stability remains a critical bottleneck for the transition of polymer thermoelectrics into practical applications. Notably, operational stability is a multifaceted requirement. While the fundamental ambient and thermal stability of thermoelectric polymers, governed by electronic factors such as LUMO level positioning and oxidation resistance, is discussed in detail in Section 6, true longevity in a functional device extends beyond these molecular parameters. For example, in a wearable patch that is powered by body heat, the device is subjected to persistent mechanical deformation and repetitive thermal cycling, both of which can severely degrade the operational lifespan. On one hand, the key features for most high‐performance thermoelectrics are their high crystallinity and tight packing for efficient intermolecular charge transport. On the other hand, high‐crystalline polymers are often brittle. Consequently, it is essential to design polymers that are not only crystalline enough (or in other words, aggregating enough) to maintain high charge mobility but also amorphous enough to accommodate strain without fracture. Recently, near‐amorphous polymers have emerged as a promising solution, demonstrating excellent charge transport properties despite substantially disordered morphology [182]. Most recently, Müller et al. reported a promising strategy to resolve the stiffness‐conductivity trade‐off by utilizing the role of dopant counterions [275]. By rational doping glycolated thienothiophene‐based copolymers, the electrical and mechanical properties can be effectively decoupled. Specifically, while highly oxidized films maintain a comparable electrical conductivity regardless of counterion size, the choice of counterion significantly shifts the sub‐glass transition temperature (Figure 24C). This enables the room‐temperature elastic modulus to be tuned without sacrificing electronic performance. This work suggests that charge transport, which is governed by the backbone, and mechanical relaxation, which is governed by side chains, can be addressed independently, thus opening new avenues for sustainable bioelectronics where both rigid and soft components could potentially be fabricated from the same polymer matrix [275].

Moreover, interfacial fatigue poses a substantial threat to device longevity. For instance, the metal‐polymer junction is frequently the primary point of failure. Since thermoelectric devices are designed to undergo temperature gradients, the thermal cycling can lead to delamination at the polymer‐electrode interfaces, or cracking within the polymer matrix. Specifically, the mismatched coefficients of thermal expansion (CTE) [278, 279] between the rigid metal electrodes and the compliant polymer matrix frequently lead to delamination and the subsequent loss of electrical contact. Addressing this requires future advances in interfacial engineering, including, for instance, the innovation of conductive buffer layers, and the development of stretchable interconnects that can bridge the mechanical and thermal gap between rigid inorganic contacts and soft organic layers.

The previously discussed bottlenecks, including orthogonality constraint, CTE mismatch, and interfacial fatigue, etc., all lead to the difficulties in realizing seamless integration of polymer thermoelectric generators with other electronic components, such as transistors and supercapacitors. Beyond these hurdles, the integration is also hampered by high contact resistance at the polymer‐electrode interface [280]. Specifically, the doping process may shift the work function of the polymers, making it difficult to maintain a stable Ohmic contact over extended operation. Furthermore, the spontaneous migration of dopants from the active thermoelectric polymer layer into adjacent electronic components can cause a detrimental effect on the performance of the connected sensitive devices, like OFETs, shifting their threshold voltages and introducing undesired leakage currents. Consequently, the transition from a high‐performance polymer to a functional integrated system requires a comprehensive strategy that goes beyond materials science, necessitating synergistic advancements in interfacial chemistry, processing innovations, thermomechanical engineering, as well as more sophisticated manufacturing.

## 9 Conclusions and Outlook

Over the past several years, significant progress has been made in the development of polymer thermoelectrics, transitioning the field from empirical observations to a mechanistically driven design approach. First, the emergence of the benchmark n‐type PBFDO, along with the synthesis of several novel electron‐deficient and ambipolar polymers, is filling the long‐standing gap in high‐performance n‐type organic thermoelectrics. Emerging doping approaches, such as IEx doping, (photo)catalytic doping, light‐triggered doping, and chiral‐modulated doping, have significantly enhanced the doping efficiency and broadened the doping toolkit available for tuning electrical and thermoelectric performance. Moreover, various strategies have been developed to mitigate dopant‐induced disorder, thereby resolving the trade‐off between carrier density and charge mobility to enhance the thermoelectric performance. Notably, unconventional strategies have been developed to overcome the trade‐off between electronic and thermal properties in polymer thermoelectrics. In addition, a better understanding of stability issues has been achieved, leading to the realization of higher thermal and ambient robustness.

Bridging these fundamental breakthroughs to real‐world utility, thermoelectric polymers have been applied in various unconventional fields, including printed thermoelectrics, high‐strength textile thermoelectrics, intrinsically stretchable thermoelectric elastomers, and high‐speed biocompatible sensing platforms. Concurrently, the development of new quantitative models, e.g., the semi‐localized transport model, allows for unifying both localized and delocalized transport contributions within a single theoretical framework, thus offering a significant advancement in predicting and controlling the doping of conjugated polymers [281, 282]. Furthermore, advancements in synthetic methods are enabling the green synthesis of thermoelectric polymers [283, 284]. All these advances have ushered the field of polymer thermoelectrics into a new era.

Despite these milestones, the air‐stability issue, particularly for n‐type polymers, remains the primary bottleneck for the commercialization of polymer thermoelectrics. Consequently, major focuses and priorities in the future continue to be the design of novel electron‐deficient conjugated polymers with deeper LUMO levels, the synthesis of air‐stable n‐type dopants, and the advancement of robust doping approaches. Moreover, the path toward commercial viability necessitates a shift in focus from laboratory‐level performance to industrial‐scale integration. It is essential to address the remaining engineering hurdles of cost‐effective scalability (e.g., R2R processing), interfacial compatibility with existing electronics, and long‐term mechanical reliability under operational stress. It can be foreseen that with the rapid development of machine learning [285], especially deep learning [286], progress in polymer thermoelectrics will accelerate further. Ultimately, bridging the gap between molecular design, scalable manufacturing, and system‐level device integration will enable the widespread application of polymer thermoelectrics in the trillion‐node IoT and beyond.

## Conflicts of Interest

The author declares no conflicts of interest.

## Data Availability

The author has nothing to report.
